# 3D CT Anatomy and Morphology of the Appendicular Skeleton of European Pond Turtle (*Emys orbicularis*)

**DOI:** 10.1002/vms3.71019

**Published:** 2026-07-09

**Authors:** Omid Zehtabvar, Ali Reza Vajhi, Somaye Davudypoor, Roshanak Mokhtari, Kiana Farahani, Seyyed Hossein Modarres Tonekabony

**Affiliations:** ^1^ Anatomy Sector Department of Basic Sciences Faculty of Veterinary Medicine University of Tehran Tehran Iran; ^2^ Department of Surgery and Radiology Faculty of Veterinary Medicine University of Tehran Tehran Iran; ^3^ Veterinary Radiologist, Graduated from Faculty of Veterinary Medicine University of Tehran Tehran Iran; ^4^ Clinical Instructor, Department of Molecular and Biological Sciences College of Veterinary Medicine North Carolina State University Raleigh North Carolina USA; ^5^ DVM, Graduated from Faculty of Veterinary University of Tehran Tehran Iran

**Keywords:** appendicular skeleton, computed tomography (CT) scan, European pond turtle, micro CT scan

## Abstract

**Background:**

Turtles have special anatomical features due to their shell and the enclosure of their internal organs. One of the important differences between the anatomy of this group of reptiles and others is the skeletal structure. The European pond turtle (*Emys orbicularis*) is a species within the Emylidae family, inhabiting regions spanning Europe, Africa and Asia.

**Objective:**

This study has been designed to assess the anatomical features of the appendicular skeletal system in this species and draw comparisons with other turtle species.

**Methods:**

Computed tomography (CT) Scan Machine and Micro CT Scan Machine were used for this study. Gross anatomical studies were performed on prepared bones using the method of using insects.

**Results:**

The carpal region consists of 10 bones (5 proximal and 5 distal), whereas the tarsal region features a fused *Astragalus* and four distal bones, both following a 2.3.3.3.2 phalangeal formula. Significant sexual dimorphism is limited to the pelvic girdle, where males exhibit a more prominent pubic ridge and an ossified symphysis compared to the predominantly cartilaginous structure in females.

**Conclusions:**

The European pond turtle displays distinct anatomical characteristics compared to other turtle species, particularly aquatic ones. Notably, this turtle exhibits nails on all its manus digits, which are elongated and non‐fin‐like. In conclusion, the use of diagnostic imaging techniques, such as CT scans and micro‐CT scans, in the study of turtle skeletons has proven to be highly beneficial. These techniques enable researchers to accurately determine the direction and position of bones, even in species with complex anatomical features like turtles.

## Graphical abstract

This study has been designed to assess the anatomical features of the skeletal system in this species and draw comparisons with other turtle species. CT‐Scan Machine and Micro CT‐Scan Machine were used for this study. Gross anatomical studies were performed on prepared bones using the method of using insects.

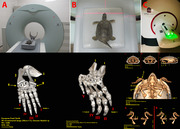

## 1 Introduction

The distinctive feature of the turtle is its shell, a protective bony structure. Consequently, this species exhibits unique skeletal anatomy, both in direct connection with shell elements, including the vertebral column and sternum, and indirect associations, impacting the appendicular skeleton. These deviations from typical reptilian anatomy are primarily attributed to the presence and form of the shell.

The distinctive locomotion patterns of this turtle, which deviate from conventional reptilian locomotion, necessitate a comprehensive examination of its skeletal system, warranting specialized evaluation methods. The European pond turtle (*Emys orbicularis*) is a species within the Emylidae family, inhabiting regions spanning Europe, Africa and Asia. Notably, segments of Iran's territory serve as a natural habitat for this species (Anderson 1972).

Computed tomography (CT) emerges as a preeminent modality for exploring the musculoskeletal and respiratory systems, both in the context of human and animal subjects. Its widespread application in scientific research underscores its efficacy in providing in‐depth insights into the anatomical intricacies and functional adaptations of diverse species, including the European pond turtle (Zehtabvar et al. 2022, 2023).

In their 2001 study, Wyneken conducted an evaluation of the skeletal system of sea turtles and made the following noteworthy findings (Wyneken 2001).

In a study conducted by Asadi Ahranjani et al. in 2016, the focus was on the radiographical and topographical features of the appendicular skeletal system in the Euphrates softshell turtle (*Rafetus euphraticus*). Their research highlighted that certain elements of the turtle's appendicular skeleton, specifically the carpal and tarsal bones, hold significance for taxonomic classification within the turtle species (Asadi Ahranjani et al. 2016).

The findings of this study indicated a taxonomic similarity between the carpal and tarsal bones of the Euphrates softshell turtle and those of the Spiny softshell turtle (*Apalone spinifera*).

In 2012, a study conducted by Bortolini et al. focused on the evaluation of the appendicular skeleton in red‐footed tortoise (*Chelonoidis carbonaria*) through both 3D reconstruction and conventional radiography.

Externally, Valente et al. emphasized the significance of two primary external landmarks: the vertebral scutes and the lateral scutes. Internally, the caudal border of the pulmonary field and bronchi were among the key internal landmarks. Furthermore, Valente et al. highlighted the significance of the coracoid bones and the acetabulum as internal landmarks.

In 2022, Hussein Yousif conducted a study that focused on the evaluation of the skeletal system of various species of turtles in the region of Iraq (Hussein Yousif 2022).

Moreover, the study by Bortolini et al. also emphasized that the pectoral girdle of *C. carbonaria* results from the fusion of the scapula and coracoid bones. Significantly, these two bones do not possess any bony connections with the shell, which is a distinguishing feature in the skeletal anatomy of this species (Bortolini et al. 2012).

Additionally, the research noted variability in the fusion of carpal and tarsal bones within *C. carbonaria*. These bones may either be fused together or remain unfused, indicating some degree of anatomical diversity within this species.

In their 2012 study, Jones et al. investigated the cranial anatomy of sea turtles and the shape of turtle skulls in the Testudines order. Their findings indicated that while suction feeding was not as well‐developed in Kemp's ridley sea turtle (*Lepidochelys kempii*) and *Caretta caretta*, both species displayed cranial adaptations conducive to forceful biting (Jones et al. 2012).

In their 2019 study, Young et al. investigated the morphological characteristics of turtles’ appendicular systems in relation to their function and phylogenetic transitions. One key finding was a positive allometric relationship between humerus bone widening and bone diameter perpendicular to the elbow joint's flexion–extension plate. Additionally, softshell turtles exhibited a positive allometric relationship between femoral diameter and body mass. This adaptation enhances their buoyancy control, facilitating effective floating in aquatic environments (Young et al. 2019).

In 2006, Valente et al. conducted a study that focused on the normal radiographic features of the cervical and coelomic structures in loggerhead sea turtle *(C. caretta*). Their research highlighted several key findings regarding the anatomical landmarks in these turtles, both externally and internally (Valente et al. 2006).

In 2007, Valente et al. examined the radiographic presentation of the appendicular skeleton in juvenile and subadult loggerhead sea turtles (Valente et al. 2007).

Studies have been done on the axial skeletal system of European pond turtle, and in them, diagnostic imaging techniques such as CT scan have been used to anatomical study, but no study has been done on appendicular skeleton in this turtle species (Zehtabvar et al. 2022, 2023). Zehtabvar et al. have also pointed out that CT scan is one of the best techniques for studying the anatomy of the skeletal system, especially by using this technique, anatomical studies can be performed on a live animal and after preparing the relevant images and after the end of anaesthesia the turtle, the animal survives. This feature is very important for studies on endangered animals (Zehtabvar et al. 2022, 2023).

Artificial intelligence (AI) is increasingly transforming veterinary anatomy education by enabling the integration of advanced technologies such as three‐dimensional modelling, virtual reality and data‐driven analytical systems, which enhance students’ understanding of complex anatomical structures and improve diagnostic accuracy through the analysis of large datasets (Choudhary and Sarkar 2025; Choudhary 2026). These AI‐supported educational approaches not only provide interactive and scalable learning environments but also rely heavily on high‐quality anatomical data and imaging resources, highlighting the importance of generating precise morphological datasets. In this context, the present study contributes to this emerging field by employing CT and micro‐CT imaging to provide detailed anatomical data on the appendicular skeleton of the European pond turtle, which can serve as a reliable foundation for future AI‐assisted educational and diagnostic applications in veterinary science.

Animal models play a fundamental role in veterinary and medical education by providing a realistic biological framework for understanding anatomical structures, surgical techniques and complex physiological interactions that cannot be fully replicated by in vitro or computational methods (Choudhary 2025). In this context, the present study applies advanced imaging techniques such as CT and micro‐CT to investigate the appendicular skeleton of the European pond turtle, contributing to anatomical education and offering a non‐invasive model for detailed skeletal analysis in veterinary education and research.

Animal anatomical models have become essential tools in veterinary education by enhancing spatial understanding, improving learning outcomes and providing ethical and accessible alternatives to cadaver‐based instruction (Choudhary and Sarkar 2025). In line with these advances, the present study utilizes CT and micro‐CT imaging to investigate the appendicular skeleton of the European pond turtle, offering a precise, non‐invasive anatomical model that supports both veterinary education and detailed morphological analysis.

Considering the absence of prior appendicular skeletal anatomical research on the European pond turtle, this study has been designed to assess the anatomical features of the appendicular skeletal system in this species and draw comparisons with other turtle species. Given the ecological and natural resource significance of the European pond turtle, our study employs non‐invasive research methods to minimize harm.

## 2 Materials and Methods

We utilize CT, a highly effective technique for studying skeletal anatomy, without the necessity of euthanizing any turtles.

### 2.1 Individuals

This study encompasses a cohort of 10 mature European pond turtles (*E. orbicularis*), consisting of 5 males and 5 females. These turtles exhibit a mean weight of 450 ± 45.22 g.

These turtles were captured alive from Mazandaran Province, Iran, with the coordination of the Environmental Protection Organization. These turtles were anesthetized for the study, and after imaging and complete recovery, they were kept in suitable reptile conditions for a week and then released back into the wild in their original location. It should be noted that no turtles were killed in the process and all were released completely healthy at the original location.

Five turtle cadavers from Tehran veterinary clinics that died due to problems unrelated to the skeletal system were used for gross anatomy and micro‐CT scan studies. These cadavers were sourced from private veterinary clinics located in Tehran, Iran and underwent both micro‐CT scanning and bone collection procedures as part of the research (Zehtabvar et al. 2022, 2023).

The turtles were relocated to the Department of Anatomy at the Faculty of Veterinary Medicine, University of Tehran. To help them acclimate and readjust to their natural environment, they were housed in a specialized reptile enclosure for 1 week. During this period, the turtles were nourished with a diet consisting of whole Kilka fish, Black Sea sprat (Zehtabvar et al. 2022, 2023).

Regarding the five cadavers used for the gross anatomy study, it should be noted that three of the cadavers were traumatic lesions and had been delivered to the veterinary clinic at the time of referral. The other two cadavers were euthanized specimens that had been brought to the clinic due to respiratory problems, but due to the advanced nature of these problems, they were euthanized with the permission of the turtle owner. It should be noted that we had no role in these processes and only received the cadavers from the clinics for our studies. In general, it should be said that no turtles were killed for this study. Of these five bodies, two were male and three were female.

This study was a DVM thesis and all experimental procedures were approved by the Faculty of Veterinary Medicine, University of Tehran (Registration number: 4011 30704/6/10).

### 2.2 CT and Micro‐CT

This phase of the study was conducted within the Small Animal Radiology Department at the Faculty of Veterinary Medicine, University of Tehran. To facilitate the CT scan procedures, the turtles underwent sedation via intramuscular injection, with Ketamine administered at a dosage of 25 mg/kg and diazepam at 1 mg/kg (Zehtabvar, Vajhi, et al. 2026).

Images were taken using a Somatom Spirit II CT Scan Machine developed by Siemens Co. CT images were transversely taken to the axis of the vertebral column. For the reconstruction process, we utilized dedicated Syngo MMWP VE40A software. The selected reconstruction algorithm was the osseous‐shaded‐vp pattern.

CT scans were conducted in two positions, the first scan was performed with the head, neck and appendicular skeletal system flexed inside the shell. The second scan was conducted with these anatomical structures extended outside the shell.

The technical parameters for our imaging protocol were as follows (Zehtabvar et al. 2022, 2023):

- Rotation time: 1 s
- Slice thickness: 1 mm
- Reconstruction interval: 0.5–1 mm
- Pitch: 1
- X‐ray tube potential: 120 kV
- X‐ray tube current: 130 mA

**FIGURE 1:**
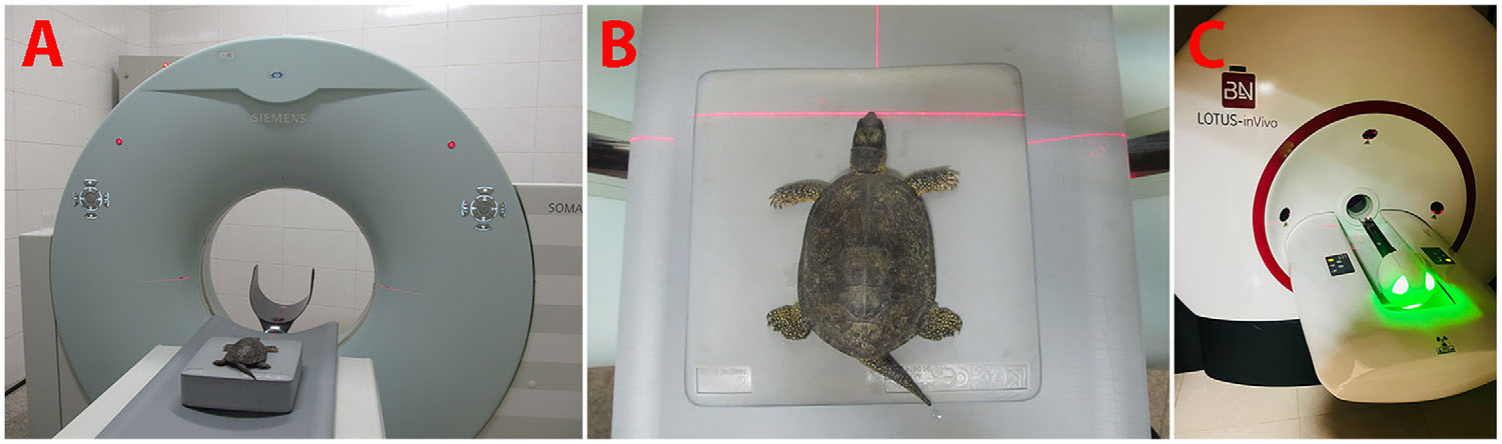
The positioning of turtle for CT scan imaging (A and B), and positioning of manus and pes of the turtle for micro‐CT scan imaging (C).

To accommodate the micro‐CT imaging process, the manus and pes of the cadavers were carefully detached from the body. The micro‐CT scans were conducted at the Preclinical Core facility (TPCF) of Tehran University of Medical Sciences (Figure 1).

In our study, we employed an in vivo x‐ray micro‐CT scanner known as LOTUS inVivo, developed by Behin Negareh Co., Tehran, Iran. This state, located at TPCF of Tehran University of Medical Sciences, features a cone beam micro‐focus x‐ray source and a flat panel detector.

The technical parameters for our imaging protocol were as follows:

- Slice thickness: 25 µm
- Magnification factor: 1.7
- Frame exposure time: 0.25 s
- X‐ray tube potential: 80 kV
- X‐ray tube current: 80 µA

The entire scanning process had a total duration of 17 min. All protocol settings and processes were meticulously controlled using LOTUS inVivo‐ACQ software. The acquired 3D data underwent reconstruction using the LOTUS inVivoREC software, employing a standard Feldkamp, Davis, Kress (FDK) algorithm for this purpose. This comprehensive imaging approach allowed us to obtain high‐resolution 3D representations of the examined bone structures.

### 2.3 Gross Anatomical Study

The skeletons of the aforementioned five cadavers, which had been euthanized for reasons unrelated to musculoskeletal issues, were carefully extracted following the removal of internal organs using *Tenebrio molitor* beetles. The beetles were maintained at a temperature of 21°C and a relative humidity of 70% to facilitate the decomposition process.

Once the skeletons were separated from the surrounding tissues by the action of the beetles, they underwent a process of bleaching and fat removal using a hydrogen peroxide solution. This procedure ensured that the skeletons were thoroughly cleaned and prepared for further analysis.

Following the preparation of the skeleton, detailed photographs were captured using an Olympus SZX12 stereo microscope equipped with an ASP‐CellPad E digital camera (Zehtabvar et al. 2022, 2023). In naming the structures in this article, it should be noted that in Herpetology some terms are traditionally used, so terms outside of NAV are also observed, but an attempt has been made to use the equivalents in NAV in parentheses.

Regarding the five cadavers used for the gross anatomy study, it should be noted that three of the cadavers were traumatic lesions and had been delivered to the veterinary clinic at the time of referral. The other two cadavers were euthanized specimens that had been brought to the clinic due to respiratory problems, but due to the advanced nature of these problems, they were euthanized with the permission of the turtle owner. It should be noted that we had no role in these processes and only received the cadavers from the clinics for our studies. It should be noted that some of the comparisons presented regarding the size of structures, especially the comparison of males and females, which are presented in Section 3, such as the comparison of the size of tubercles, are descriptive of observations made on the specimens and morphometric measurements have not been performed.

## 3 Results

**FIGURE 2:**
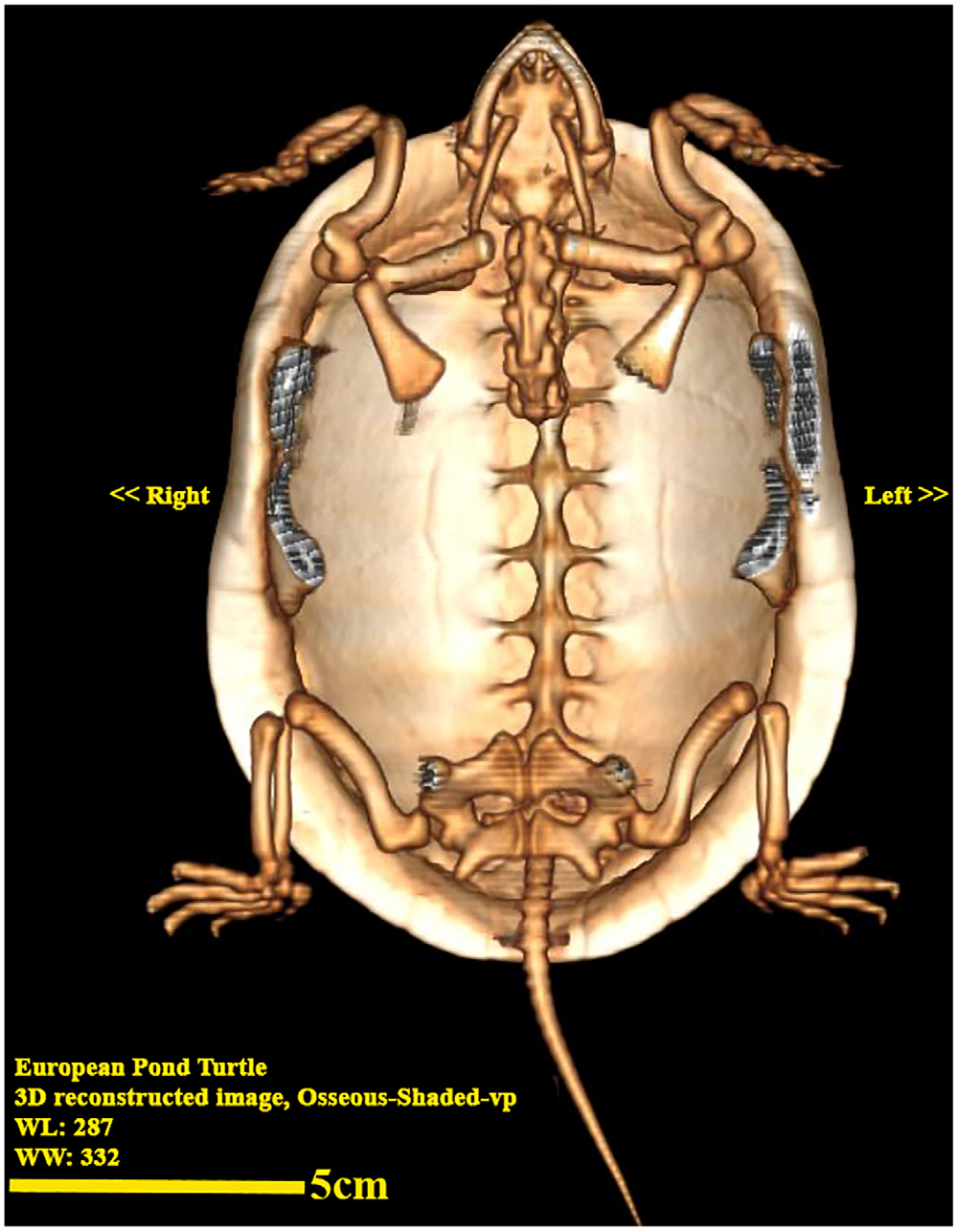
Ventral view of the skeleton of the European pond turtle, 3D reconstructed CT scan image and osseous‐shaded‐vp pattern (The plastron has been removed), the 3D clip of this figure can also be seen.

In this section, we present a comprehensive overview of the information gathered from the study, including the analysis of the prepared skeletal samples, 3D reconstructed CT scan images and micro‐CT scan images. Figure 2 and Supporting Information S1 provide a visual representation of the positioning of various components of the appendicular skeleton, which we will discuss in detail below.

### 3.1 Forelimb Bones (Ossa Membri Thoracici)

The pectoral girdle extended from a point slightly cranial to the first rib and reached the head of the third rib. It primarily consisted of the scapula bone, inclusive of its acromion process (Processus acromialis) and the coracoid bone (In this context, it's important to clarify that the term ‘rib’ refers to the costal head in this article).

**FIGURE 3:**
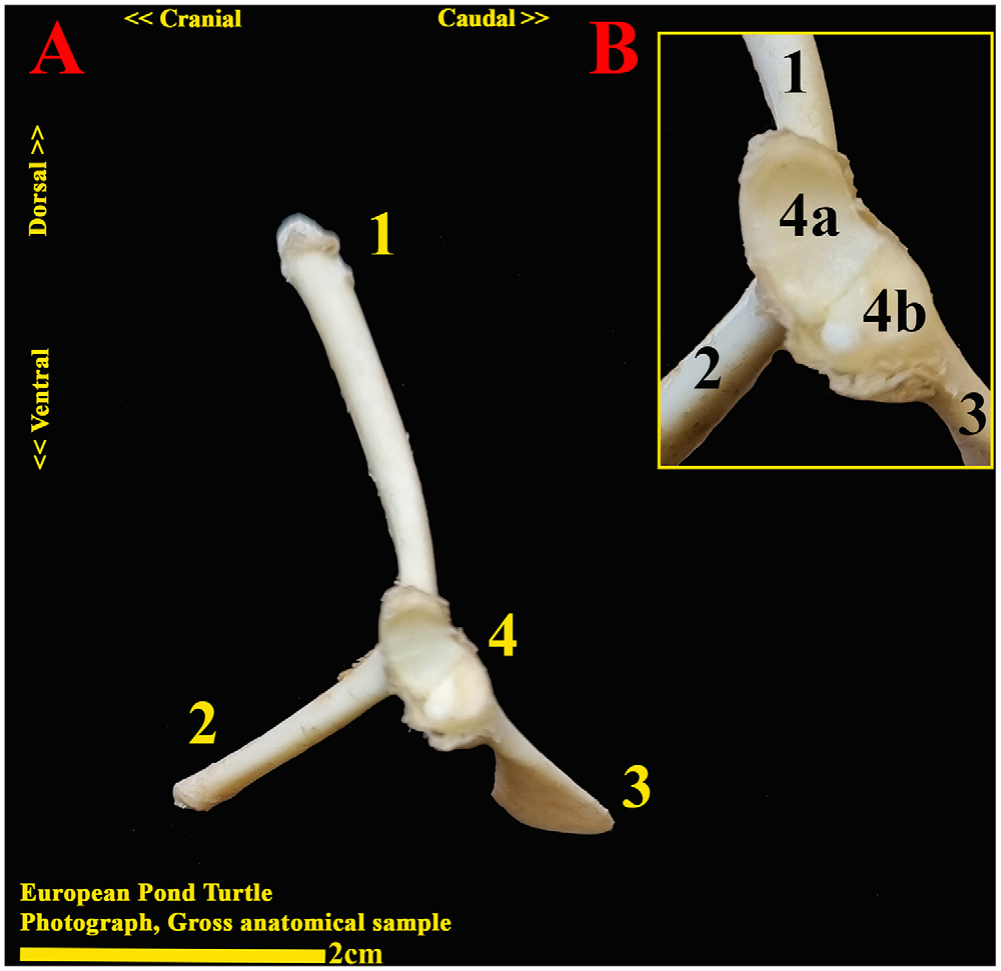
Craniolateral view of the left pectoral girdle of the European pond turtle, photograph of the gross anatomical sample, (A) general view of the structure with lower magnification, (B) closer view of glenoid cavity with higher magnification. 1. Scapula, 2. acromion process, 3. coracoid and 4. glenoid cavity (4a. scapular part, 4b. coracoid part).

The boundary between the scapula and coracoid bones was distinctly marked within the glenoid cavity. This cavity was positioned cranially and laterally (craniolateral) and was divided into two unequal parts by a demarcating line (Figure 3). The smaller part of the cavity was associated with the coracoid bone, forming the ventral aspect of this cavity. This cavity exhibited a bean‐shaped morphology (Figure 3).

Conversely, the larger part of the glenoid cavity was a result of the combined structure formed by the scapula and the acromion process. It is noteworthy that the scapula bone and the acromion process were integrated into a unified anatomical entity. The boundary delineating the two portions of the glenoid cavity, which was shaped by these structures, can be observed in Figure 3.

**FIGURE 4:**
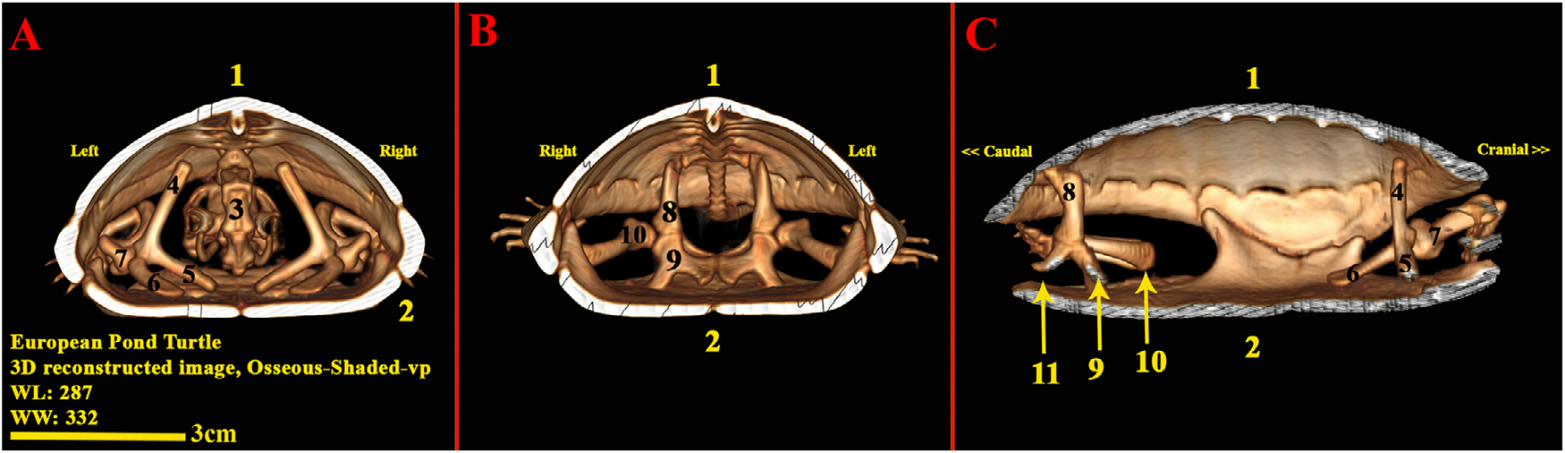
3D reconstructed CT scan images of the skeleton of the European pond turtle, osseous‐shaded‐vp pattern (The plastron has been removed), (A) transverse section, caudal view, (B) transverse section, cranial view, (C) left sagittal section, medial view. 1. Carapace, 2. plastron, 3. cervical vertebrae, 4. scapula, 5. acromion process, 6. coracoid, 7. humerus, 8. ilium, 9. pubis, 10. femur and 11. ischium.

In terms of their positioning, the coracoid bone occupied the caudomedial position, whereas the acromion process was located in the craniomedial position. The scapula bone was situated in the dorsocranial direction (Figure 4).

**FIGURE 5:**
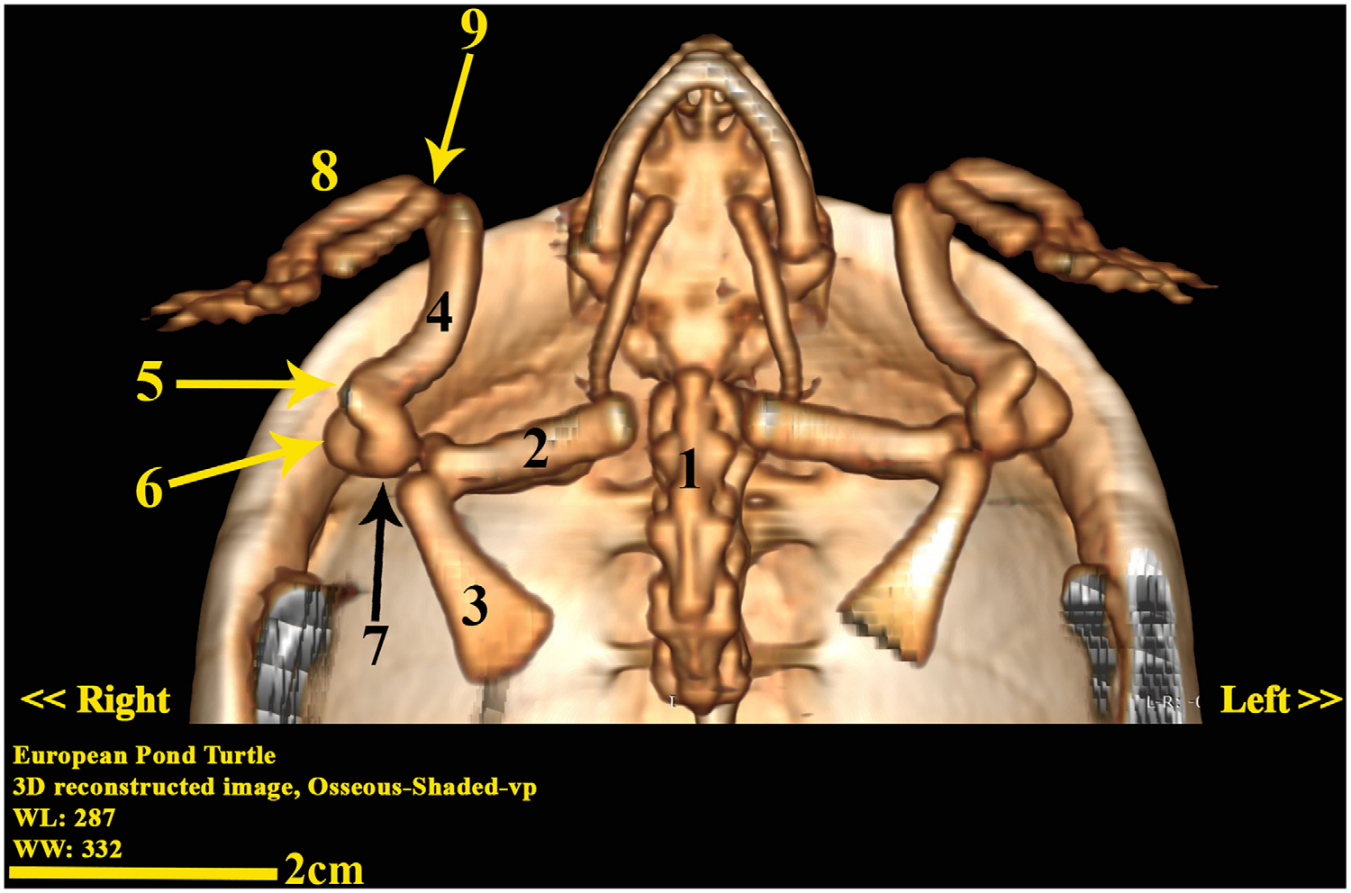
Ventral view of the skeleton of the European pond turtle, 3D reconstructed CT scan image and osseous‐shaded‐vp pattern (The plastron has been removed). 1. Cervical vertebrae, 2. acromion process, 3. coracoid, 4. humerus, 5. ventral tubercle, 6. dorsal tubercle, 7. humeral head, 8. ulna and 9. radius.

It is important to note that both the coracoid bone and acromion process exhibited a ventrally inclined orientation and were anatomically connected to parts of the plastron (Figure 5). Furthermore, the scapula was linked to a portion of the carapace at its distal end.

**FIGURE 6:**
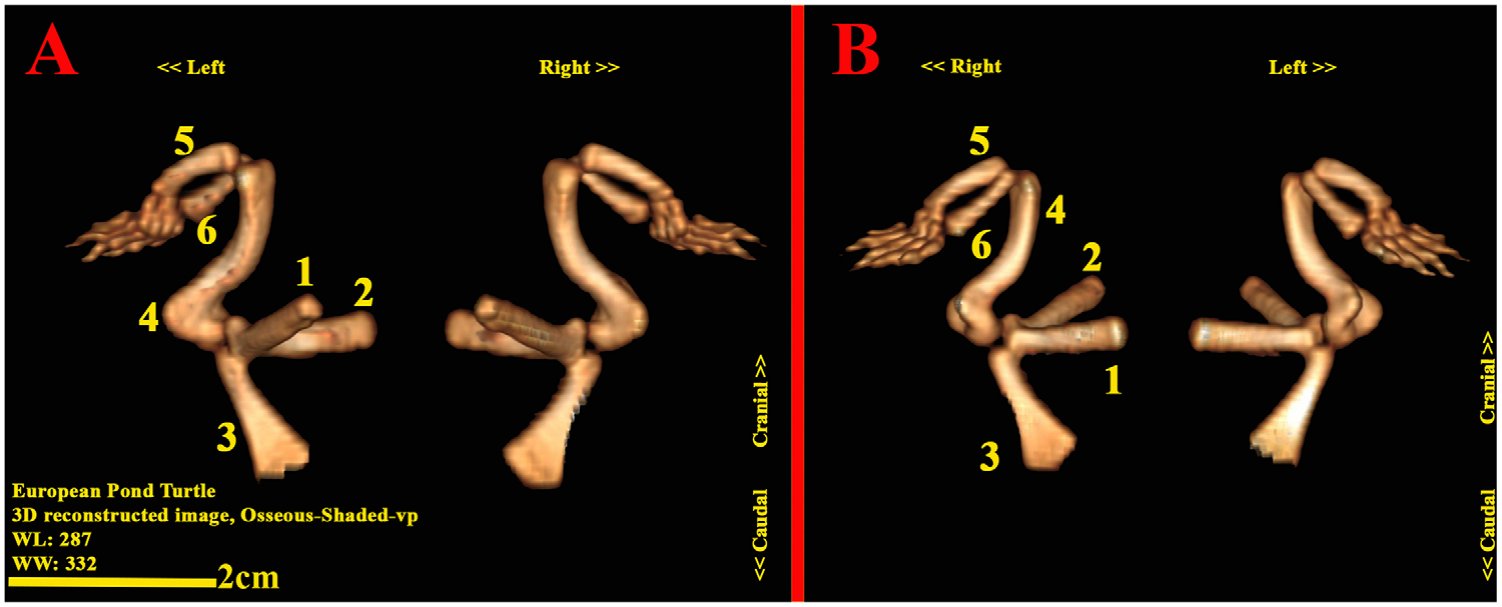
3D reconstructed CT scan images of the skeleton of the European pond turtle, osseous‐shaded‐vp pattern (The carapace and plastron has been removed), (A) dorsal view, (B) ventral view. 1. Scapula, 2. acromion process, 3. coracoid, 4. humerus, 5. ulna and 6. radius.

Describing their individual shapes, the coracoid bone took on a triangular and broad configuration, whereas the scapula bone displayed a rod‐shaped appearance. The acromion process, on the other hand, had a nearly rod‐like structure, with a slight widening towards its distal end (Figure 6).

In the area of the brachium (stylopodium), an elongated humerus bone with a slight S‐shaped curvature of its body was observed. The body of the bone was nearly three‐sided near the proximal part of the bone and gradually widened towards the distal end. The articular surface in the proximal part of the humerus was found to be wider than the glenoid cavity. At the proximal end, on both sides, two non‐articulated tubercles, namely, the dorsal and ventral tubercles, were noted, with the dorsal tubercle being the larger of the two. The humerus bone's body was delineated by a dorsal border and a ventral border. Due to the change in orientation of the humerus bone structures when the turtle stood on its limb, the dorsal tubercle could also be called the medial tubercle and the ventral tubercle could also be called the lateral tubercle.

In this region, two surfaces, namely, the lateral and medial surfaces, were identified. The medial surface exhibited prominence, whereas the lateral surface appeared depressed. It is noted that the medial surface faced cranially, whereas the lateral surface faced caudally.

**FIGURE 7:**
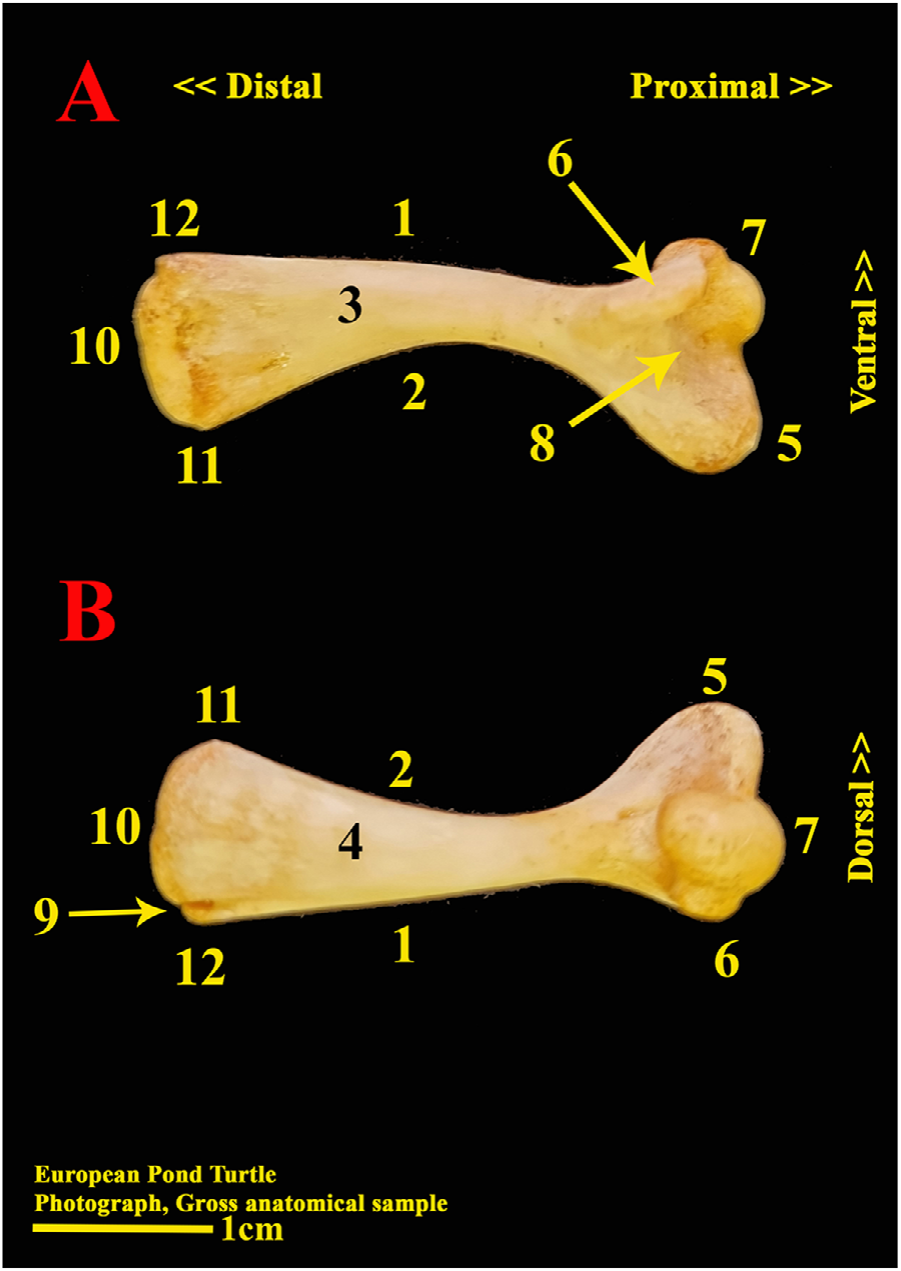
Photograph of the gross anatomical sample of the right humerus of the European pond turtle, (A) lateral view, (B) medial view. 1. Ventral border, 2. dorsal border, 3. lateral surface, 4. medial surface, 5. dorsal tubercle, 6. ventral tubercle, 7. humeral head, 8. intertubercular fossa, 9. radial groove, 10. radioulnar articular surface, 11. dorsal epicondyle and 12. ventral epicondyle.

The dorsal border displayed a more pronounced arch and concavity, in contrast to the ventral border, which had a smoother contour than the dorsal border. Additionally, in the lateral region of the proximal end of the bone, a fossa was situated between the dorsal and ventral ridges. This particular fossa was referred to as the intertubercular fossa (Figure 7).

As a result of the S‐shaped curvature of the bone's body, the articular surface of the proximal part of the bone (head) faces medially. This orientation was observed when the limb was extended outside the shell, as the extension of the bone inclined cranially. Conversely, the articular surface of the distal part was inclined laterally, and this inclination occurred when the limb was extended outside the shell, as the extension of the bone inclined caudally.

The distal part of the bone exhibited significant widening, featuring a broad articular surface that exceeded the combined size of the articular surfaces at the ends of the proximal radius and ulna. On the medial surface, near the ventral edge of the distal extremity, a groove was present, named the radial groove due to its proximity to the articulation site of the radius bone (Figure 7).

At the distal extremity, two epicondyles were observed on both sides. One of these was referred to as the dorsal epicondyle, whereas the other was termed the ventral epicondyle. It is noteworthy that these two structures were more clearly visible in micro‐CT imaging (Figures 7 and 9).

As outlined in Section 2, the CT scan images were acquired in two distinct modes, with the criteria for naming different anatomical parts based on the mode where the appendicular organs, head and neck were contained within the shell space. However, it is essential to note that this study also addresses the changes in the positioning of appendicular organs when they are extended outside the shell.

Remarkably, the shoulder girdle (Cingulum membri thoracici) remained unchanged during the process of limb extension. When the forelimb extended to support the turtle's weight, the humerus was oriented in such a way that the medial surface inclined cranially while the lateral surface inclined caudally. Consequently, in this scenario, the dorsal border inclined laterally, and the ventral border inclined medially. Additionally, the humeral head exhibited an inclination towards the cranial direction.

**FIGURE 8:**
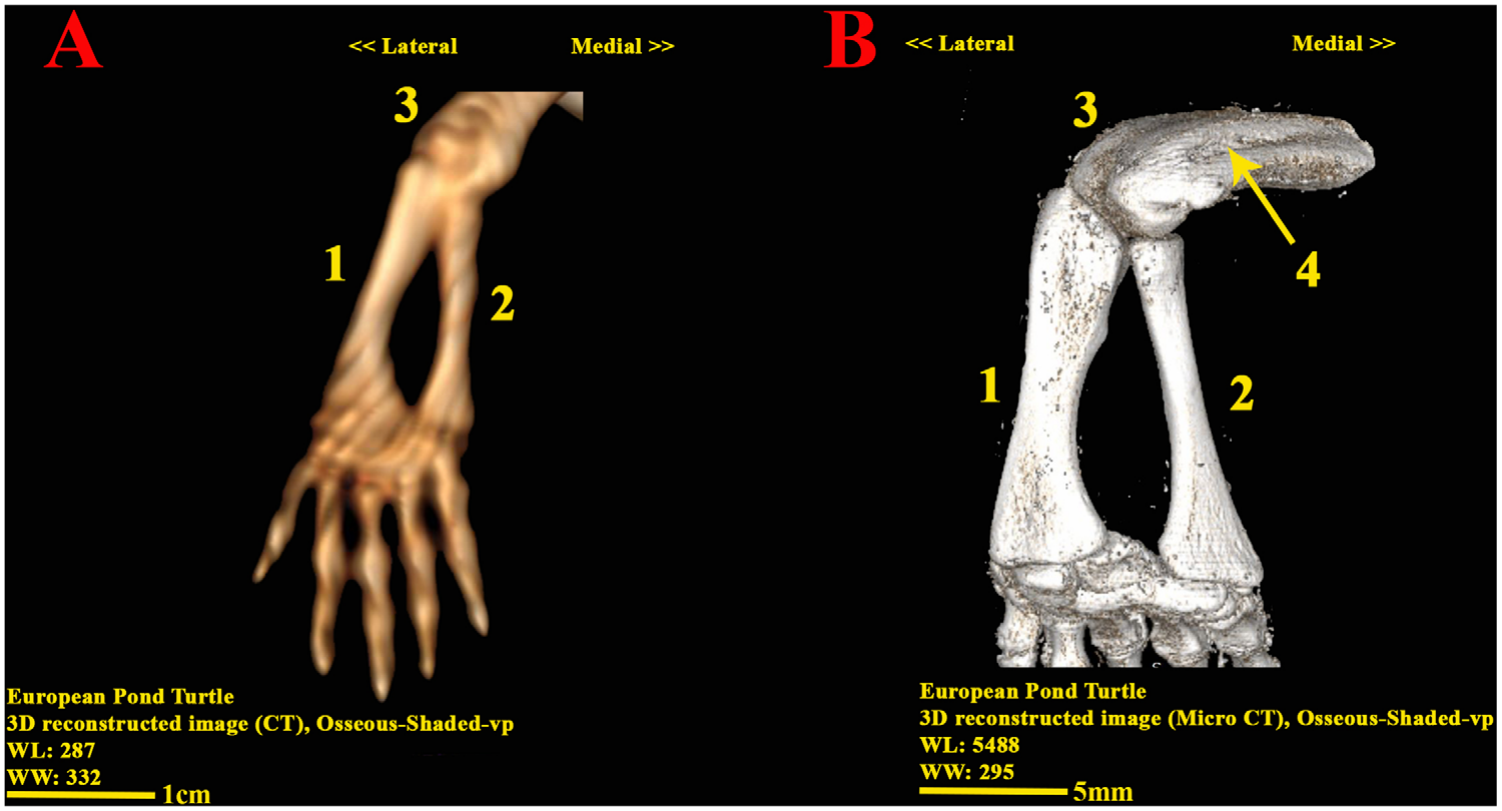
3D reconstructed CT scan images of the skeleton of the right manus, antebrachium and brachium of the European pond turtle, osseous‐shaded‐vp pattern, (A) CT, (B) micro CT. 1. Ulna, 2. radius, 3. humerus and 4. radial groove.

The radius and ulna constitute the skeleton of the antebrachium (zeugopodium). Although their proximal extremities are interconnected via periarticular soft tissues and joint structures, the distal ends remain distinct entities, each articulating independently with its respective carpal bones. A significant interosseous space is observed between the shafts of these two bones (Figure 8).

Both bones exhibited a broad structure, with the ulna's body being wider than that of the radius. The bodies of these bones displayed a narrower midsection and gradually widened at both the proximal and distal ends. The ulna bone occupied a more dorsal and lateral position compared to the radius.

**FIGURE 9:**
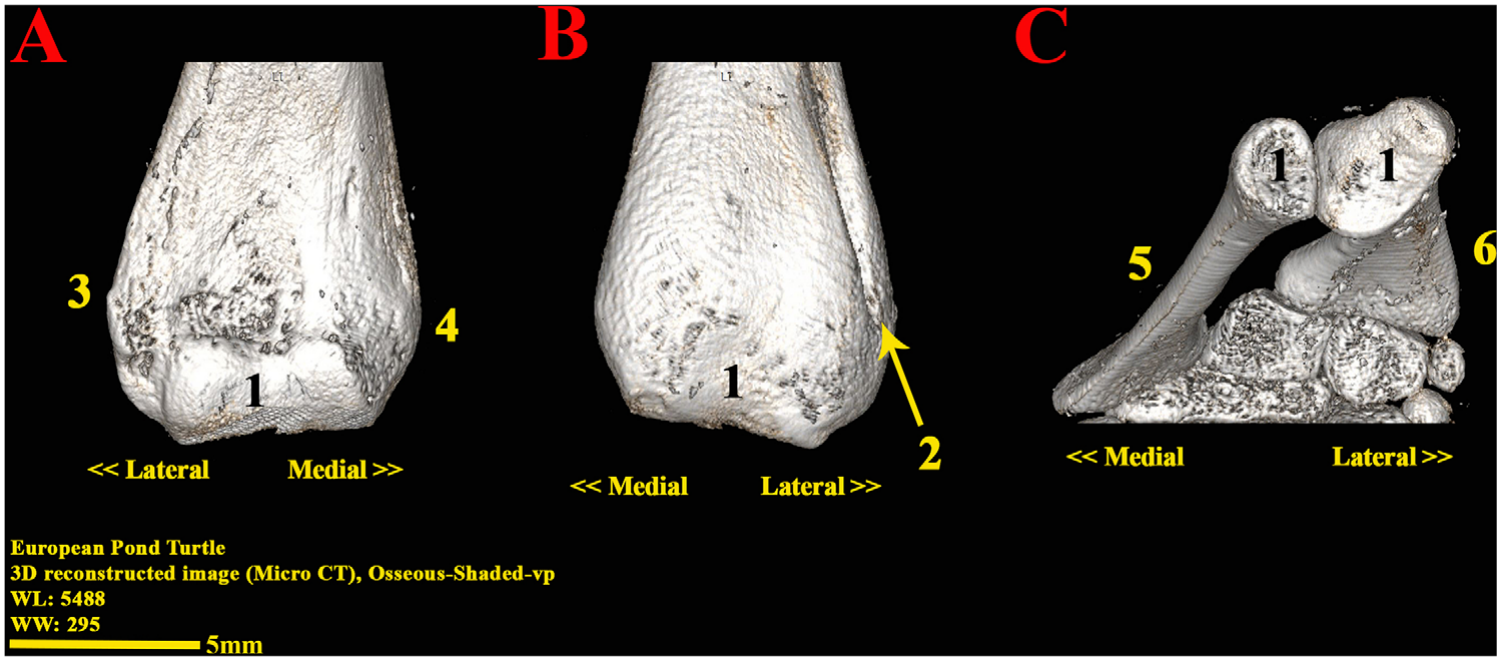
3D reconstructed micro CT scan images of the skeleton of the right humerus, radius and ulna of the European pond turtle, osseous‐shaded‐vp pattern, (A) distal extremity of the humerus, lateral view, (B) distal extremity of the humerus, medial view, (C) proximal extremity of the radius and ulna, proximal view. 1. Articular surface, 2. radial groove, 3. ventral epicondyle, 4. dorsal epicondyle, 5. radius and 6. ulna.

At the proximal extremity of both bones, there existed an articular surface designed for articulation with the humerus. Notably, the articular surface of the ulna was larger compared to that of the radius (Figure 9).

At the proximal extremity, both bones collectively formed a shared articular surface intended for articulation with the humerus bone. In this arrangement, the larger portion of the articular surface belonged to the ulna, whereas the smaller part was attributed to the radius. However, it is worth noting that the combined area of these two articular surfaces was still smaller than the surface located at the distal end of the humerus.

**FIGURE 10:**
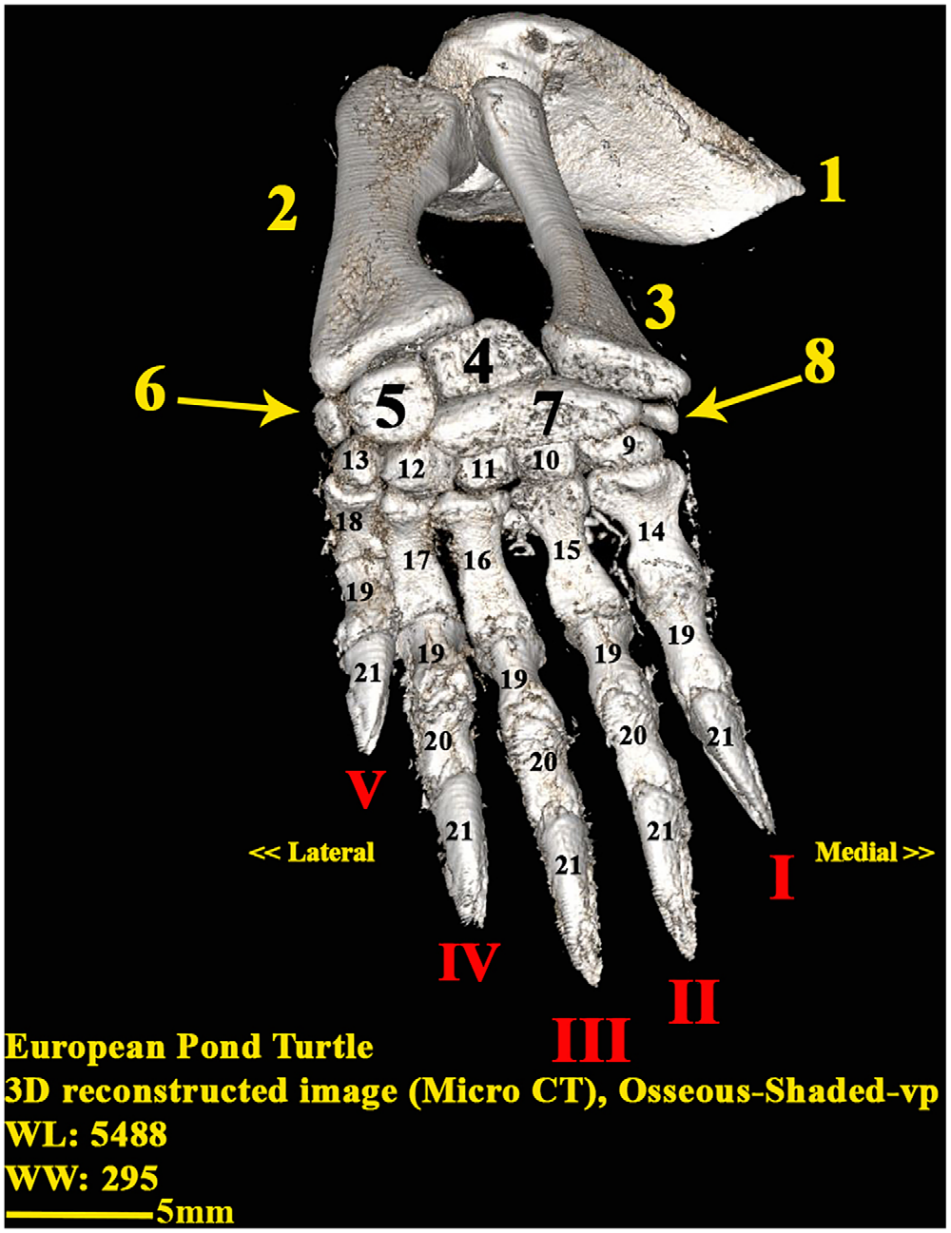
3D reconstructed micro CT scan images of the skeleton of the right humerus, radius, ulna and manus of the European pond turtle, osseous‐shaded‐vp pattern, dorsal view of the manus shown, finger numbers are marked with Roman numerals, the 3D clip of this figure can also be seen. 1.  Humerus, 2.  ulna, 3.  radius, 4.  radiale, 5.  ulnare, 6.  pisiform, 7.  centrale, 8.  sesamoid bone, 9.  1st distal carpal, 10.  2nd distal carpal, 11.  3rd distal carpal, 12.  4th distal carpal, 13.  5th distal carpal, 14.  1st metacarpal, 15.  2nd metacarpal, 16.  3rd metacarpal, 17.  4th metacarpal, 18.  5th metacarpal, 19.  1st phalanx, 20.  2nd phalanx and 21.  3rd phalanx.

Regarding the distal end of the radius, it extended further distally. The radius bone was articulated with the radiale (Os carpi radiale) and centrale bones, whereas the ulna bone was articulated with the ulnare (Os carpi ulnare) and radiale bones of the proximal carpal row (Figure 10, Supporting Information S2).

As evident from the CT scan images, such as Figure 8, the structure of the manus region (autopodium) was not clearly discernible. Therefore, the information provided in this section is derived from the analysis of micro‐CT scan images and bone samples that were isolated during the gross anatomy examination.

Within this region, we observed the presence of carpal bones, metacarpals and phalanges. The carpal bones were arranged into two distinct rows, namely, the proximal and distal carpal rows.

**FIGURE 11:**
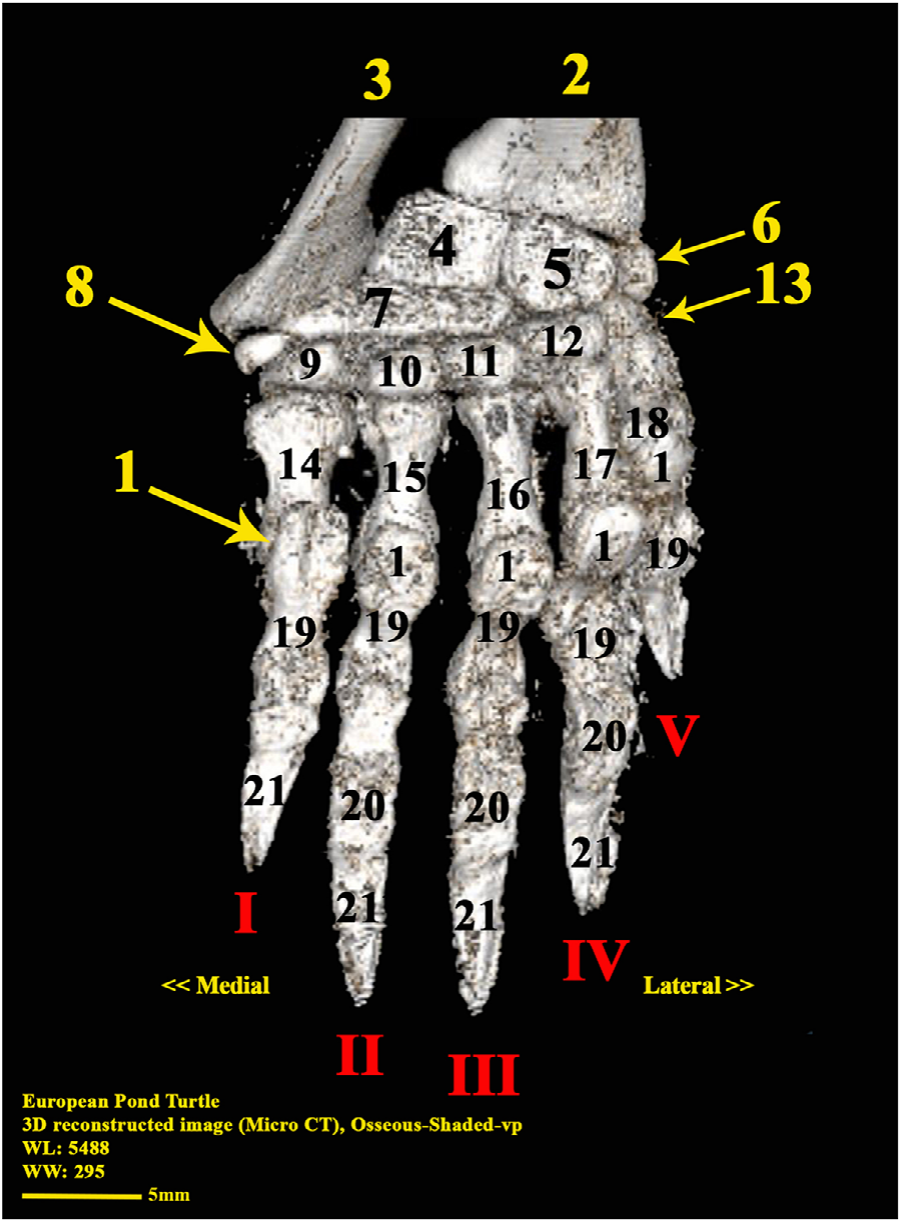
3D reconstructed micro CT scan images of the skeleton of the right radius, ulna and manus of the European pond turtle, osseous‐shaded‐vp pattern, palmar view of the manus shown, finger numbers are marked with Roman numerals, the 3D clip of this figure can also be seen. 1.  Prominence of the palmar surface of the phalanx, 2.  ulna, 3.  radius, 4.  radiale, 5.  ulnare, 6.  pisiform, 7.  centrale, 8.  sesamoid bone, 9.  1st distal carpal, 10.  2nd distal carpal, 11.  3rd distal carpal, 12.  4th distal carpal, 13.  5th distal carpal, 14.  1st metacarpal, 15.  2nd metacarpal, 16.  3rd metacarpal, 17.  4th metacarpal, 18.  5th metacarpal, 19.  1st phalanx, 20.  2nd phalanx and 21.  3rd phalanx.

Both the proximal and distal rows of carpal bones consisted of five bones each. The bones within the proximal row exhibited distinct size variations. Notably, the largest among them was the centrale bone (Os carpi centrale), positioned beneath the radius bone and also articulating dorsally. Adjacent to the centrale bone on the lateral side was the ulnare bone, followed by the delicate pisiform bone (Os carpi accessorium). Additionally, a small sesamoid bone was observed on the medial side of the prominent centrale bone (Figures 10 and 11).

To summarize, the proximal row of carpal bones consisted of five bones, arranged in the following order from lateral to medial: pisiform, ulnare, radiale, centrale and a sesamoid bone situated on the medial side of the centrale.

The five bones of the distal row were arranged linearly, with each of them positioned on top of a corresponding metacarpal bone. These distal carpal bones (Ossa carpi distalia) are articulated with their respective metacarpal bones.

The first distal carpal bone was proximally connected to the centrale bone and the adjacent sesamoid bone. The second and third distal carpal bones articulated with the centrale bone, whereas the fourth and fifth distal carpal bones were articulated with the ulnare bone. Notably, it is important to mention that the fifth distal carpal bone also had an articulation point with the pisiform bone (Figures 10 and 11) (Table 1).

**TABLE 1:** Autopodial formula (bones) of the European pond turtle.

Number of proximal carpal bones	Number of distal carpal bones	Number of metacarpal bones	Manus phalangeal formula^a^
5	5	5	2.3.3.3.2

Number of proximal tarsal bones	Number of distal tarsal bones	Number of metatarsal bones	Pedis phalangeal formula^a^
1	4	5	2.3.3.3.2

^a^ Medial to lateral.

Within the manus region, five metacarpal bones were identified. Notably, the extremities of these metacarpal bones were broader than their midsections. Furthermore, there was a gradual decrease in width from the first metacarpal bone to the fifth metacarpal bone. The third metacarpal bone, in particular, was longer than the others.

The proximal extremity of each metacarpal bone articulated with the corresponding distal carpal bones of the same number, whereas their distal extremities formed articulations with the first joint of the fingers (phalanges) (Figures 10 and 11).

The first and fifth digits each consisted of two phalanges, whereas digits two, three and four comprised three phalanges. Notably, all five digits on one manus were characterized by the presence of an unguis on the third phalanx.

Additionally, the first phalanx of these digits exhibited a notable protrusion on the proximal part of their palmar surface. Importantly, there were no sesamoid bones observed in the vicinity of any of the phalanges (Figures 10 and 11 and Table 1). In general, when the anterior motor limb was positioned upright, structures that were described as dorsally positioned during limb extension were positioned medially, and structures that were ventrally positioned were positioned laterally.

### 3.2 Hind Limb Bones (Ossa Membri Pelvini)

The pelvic girdle (Cingulum membri pelvini) comprised two hip bones (Os coxae), which articulated at two distinct points. One articulation occurred in the cranial region between the medial edges of the two pubic bones, whereas the other took place in the caudal area between the inner edges of the two ischium bones. Each hip bone was composed of three separate bones, and their junctions were clearly defined in the form of a dentated suture. These three bones converged at a joint cavity referred to as the acetabulum.

The acetabulum assumed a triangular shape, with the apex of the triangle located ventrally. Additionally, the acetabulum was inclined laterally and cranially. The pubic bone and ischium collectively form the pelvic floor. A thyroid fenestra (foramen obturatum) was situated in the middle of the pelvis, positioned between the ilium, pubis and ischium bones.

**FIGURE 12:**
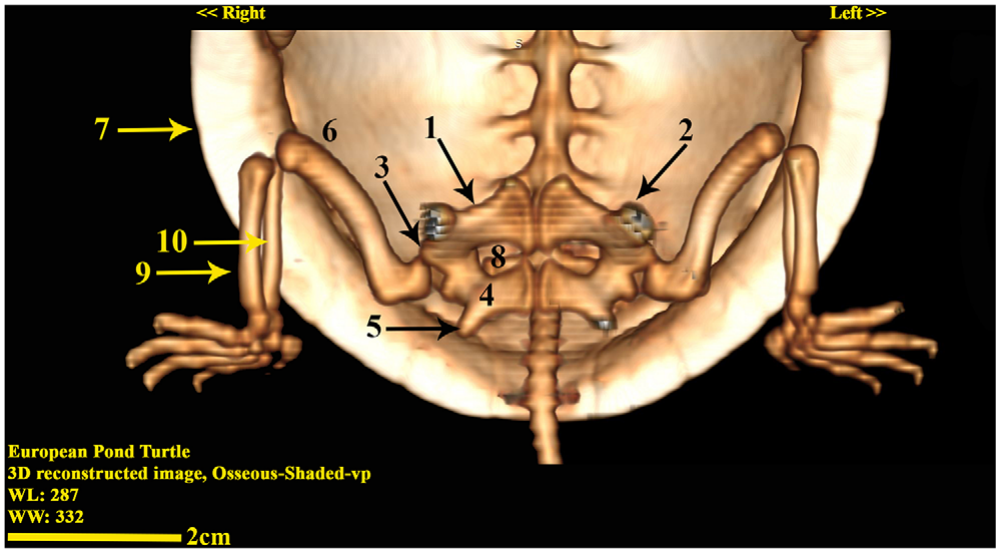
Ventral view of the skeleton of the European pond turtle, 3D reconstructed CT scan image and osseous‐shaded‐vp pattern (The plastron has been removed). 1. Pubis, 2. pubic process, 3. acetabulum, 4. ischium, 5. metischial process, 6. femur, 7. carapace, 8. thyroid fenestra, 9. tibia and 10. fibula.

**FIGURE 13:**
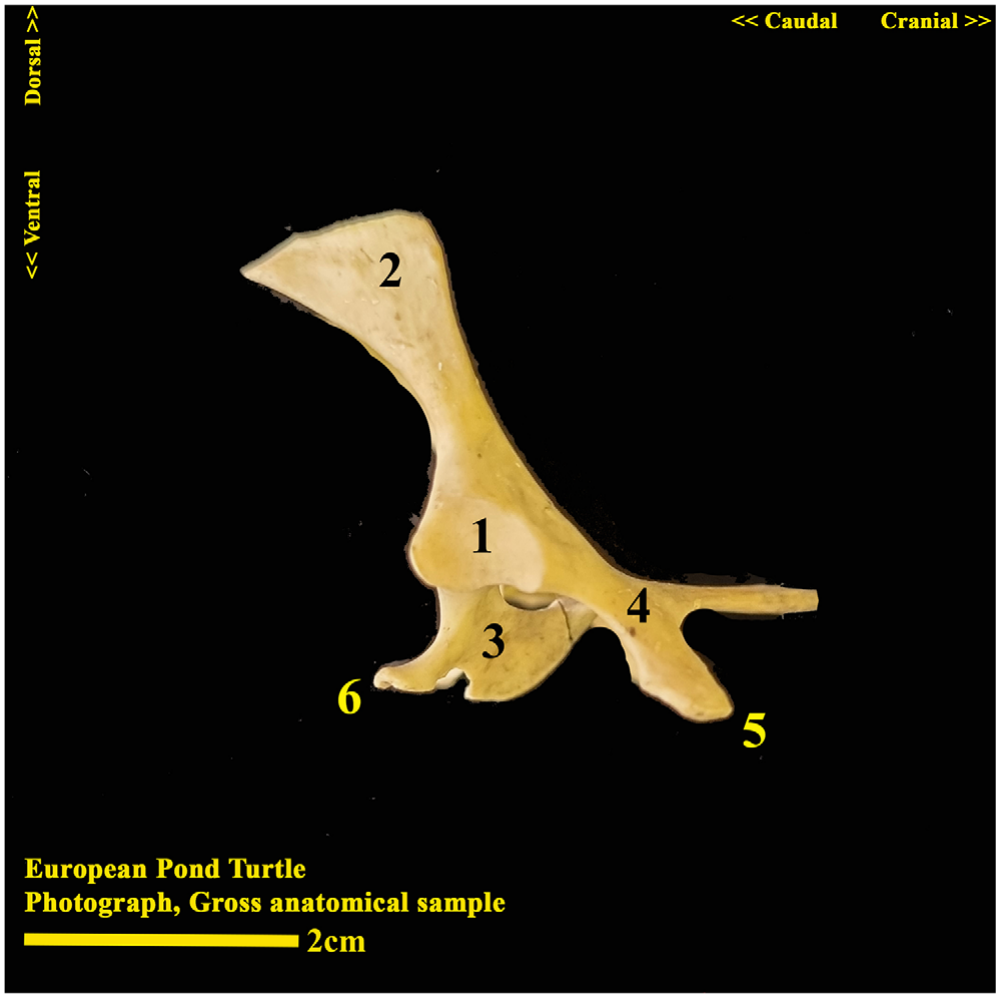
Photograph of the gross anatomical sample of the right hip bone of the European pond turtle, lateral view. 1. Acetabulum, 2. ilium, 3. ischium, 4. pubis, 5. pubic process and 6. metischial process.

Significantly, the ilium bone exhibited a caudodorsal extension (Figure 12). Furthermore, a protrusion was observed on the cranial edge of the pubic bone, oriented laterally. At the posterior edge of the ischium bone, a metischial process was also observed (Figures 12 and 13).

The femur bone was situated within the femoral area (stylopodium). It exhibited an elongated structure with a slight S‐shaped curvature along its body. At the proximal extremity, the femur featured an oval articular head, and caudal to the head, two dorsal and ventral trochanters were located caudal to the head. A fossa was positioned between these two trochanters and inclined towards the caudal aspect of the bone. The head of the bone was separated from the body by a distinct neck.

The prominent portion of the bone's body faced cranially, whereas the curved part was oriented caudally. The greater trochanter extended towards the caudal and dorsal sides of the bone. The distal half of the body was widened and had dorsal and ventral edges.

**FIGURE 14:**
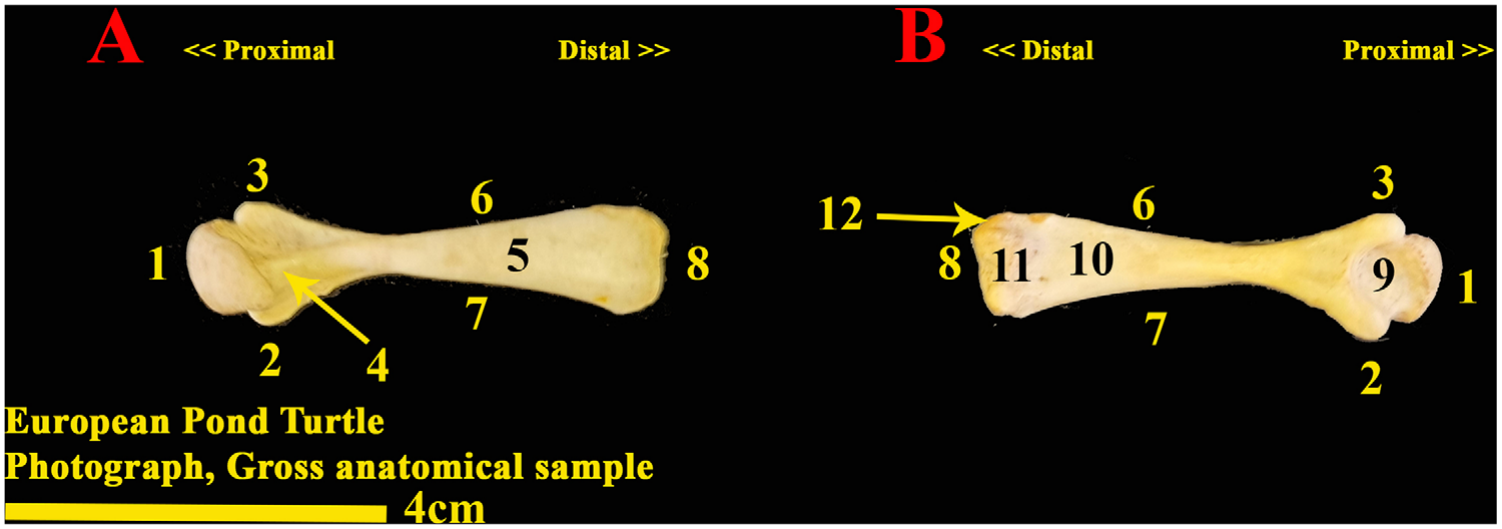
Photograph of the gross anatomical sample of the left femur of the European pond turtle, (A) cranial view, (B) caudal view. 1. Articular head, 2. ventral trochanter, 3. dosral trochanter, 4. neck, 5. cranial surface, 6. dorsal border, 7. ventral border, 8. distal extremity, 9. intertrochanteric fossa, 10. caudal surface, 11. tibial and fibular articular surface and 12. distal groove.

At the distal extremity, the bone had articular surfaces designed for articulation with the fibula and tibia bones. These two articular surfaces were nearly equal in size. Additionally, a groove was observed on the caudal surface of the distal extremity of the femur, situated close to the dorsal edge (Figure 14).

Within the crural region (zeugopodium), there were two bones: the tibia and the fibula. These bones were connected at their proximal ends. In the distal extremity, each of these bones is articulated with the *Astragalus* (talus) bone of the tarsal region through distinct joint surfaces.

The tibia bone possessed a thicker body and proximal extremity compared to the fibula. Additionally, its distal extremity was larger than the proximal extremity. On the other hand, the fibula bone had a slenderer body in comparison to the tibia (Figure 12).

The area of pes (autopodium) included tarsal bones, metatarsal bones and phalangeal bones. Within the tarsal region, there were two rows of bones. The bones in the proximal row were fused to form the single *Astragalus* (talus) bone. In the distal row, four separate bones were present.

The *Astragalus* (talus) bone was situated beneath the fibula and tibia bones and articulated with both of these bones. Distally, it articulated with the first, second and third distal tarsal bones and laterally with the fourth distal tarsal bone.

The fifth metatarsal bone exhibited a quadrangular shape, featuring a protruding articular apex used for articulation with the first phalanx of the fifth digit. This bone was positioned in the same row as the bones of the distal row of the tarsal region and had an articular surface with the fourth distal tarsal bone.

**FIGURE 15:**
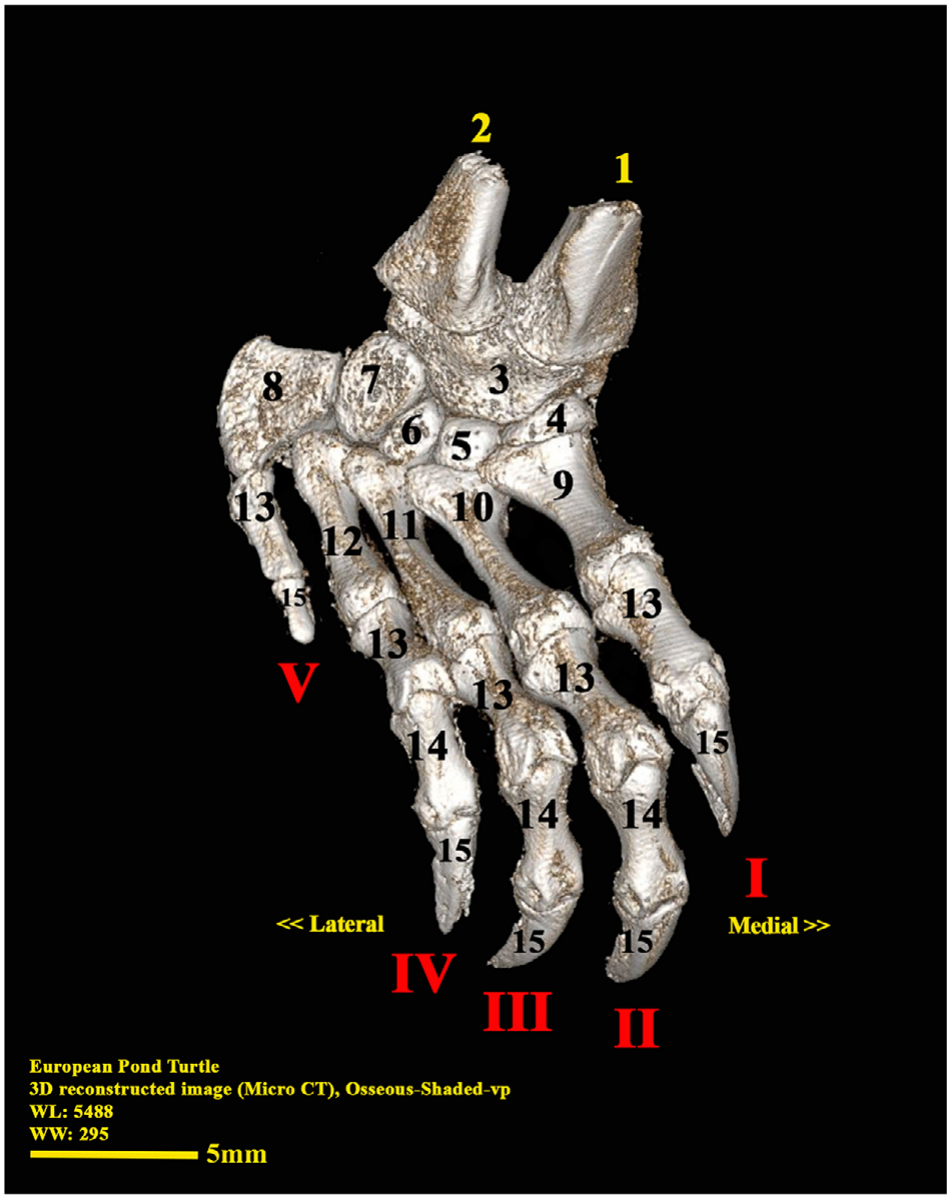
3D reconstructed micro CT scan images of the skeleton of the right pes of the European pond turtle, osseous‐shaded‐vp pattern, dorsal view of the manus shown, finger numbers are marked with Roman numerals, The 3D clip of this figure can also be seen. 1.  Tibia, 2.  fibula, 3.  *Astragalus* (talus), 4.  1st distal tarsal, 5.  2nd distal tarsal, 6.  3rd distal tarsal, 7.  4th distal tarsal, 8.  5th metatarsal, 9.  1st metatarsal, 10.  2nd metatarsal, 11.  3rd metatarsal, 12.  4th metatarsal, 13.  1st phalanx, 14.  2nd phalanx and 15.  3rd phalanx.

Digits one and five each consisted of two phalanges, whereas digits two, three and four had three phalanges. All fingers, except the fifth digit, possessed an unguis on their third phalanx. Notably, the third phalanx of the fifth digit in the pes area differed in shape from the others (Figure 15, Supporting Information S3) (Table 1).

**FIGURE 16:**
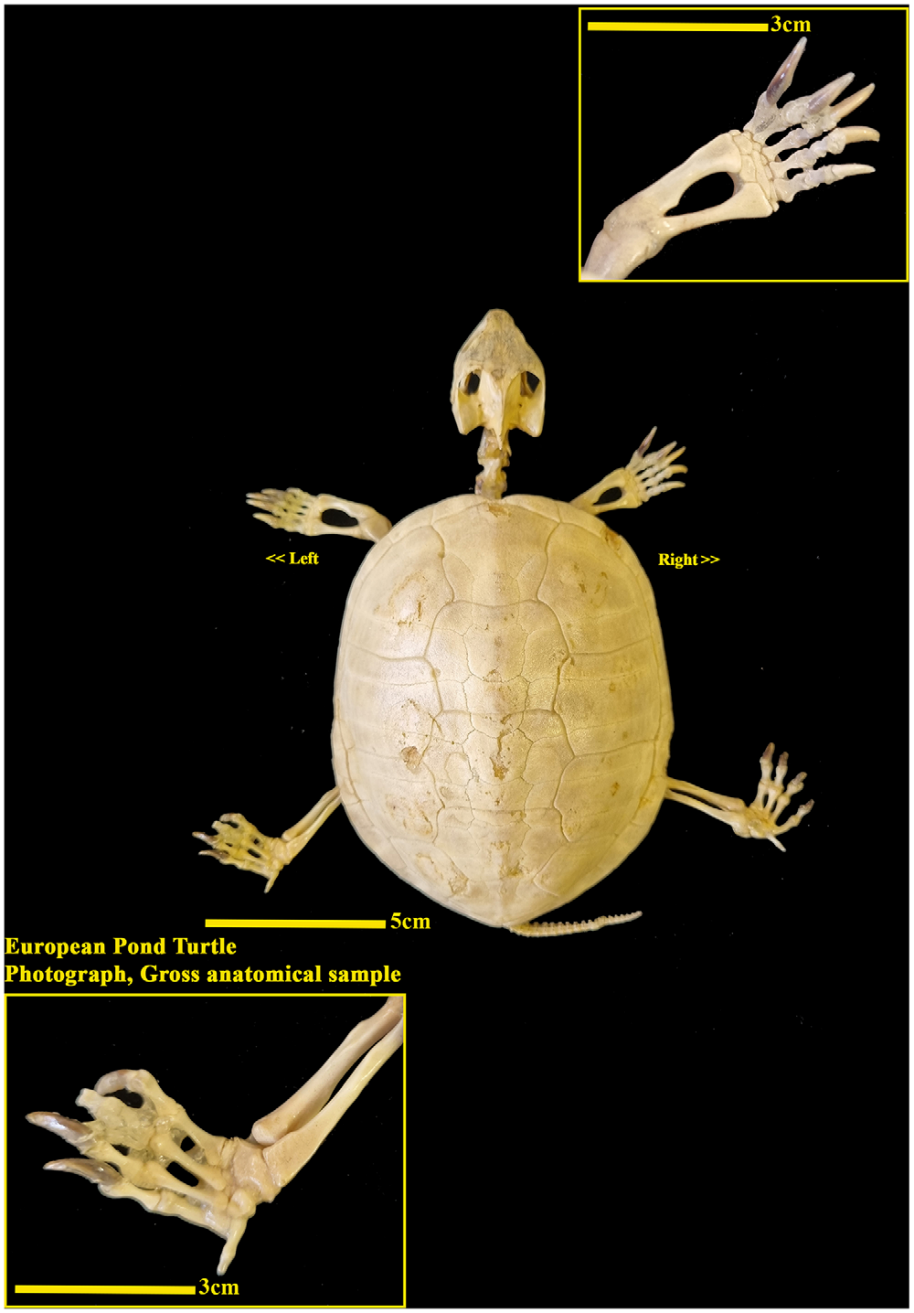
Photograph of the gross anatomical sample (complete skeleton, dorsal view) of the European pond turtle, it should be noted in this image that the manus is rotated outwards and we are viewing them from the palmar surface, and the radius and ulna have also rotated to create this position. As can be seen in the enlarged image, the third phalanx of the second toe is missing on the left pes.

**FIGURE 17:**
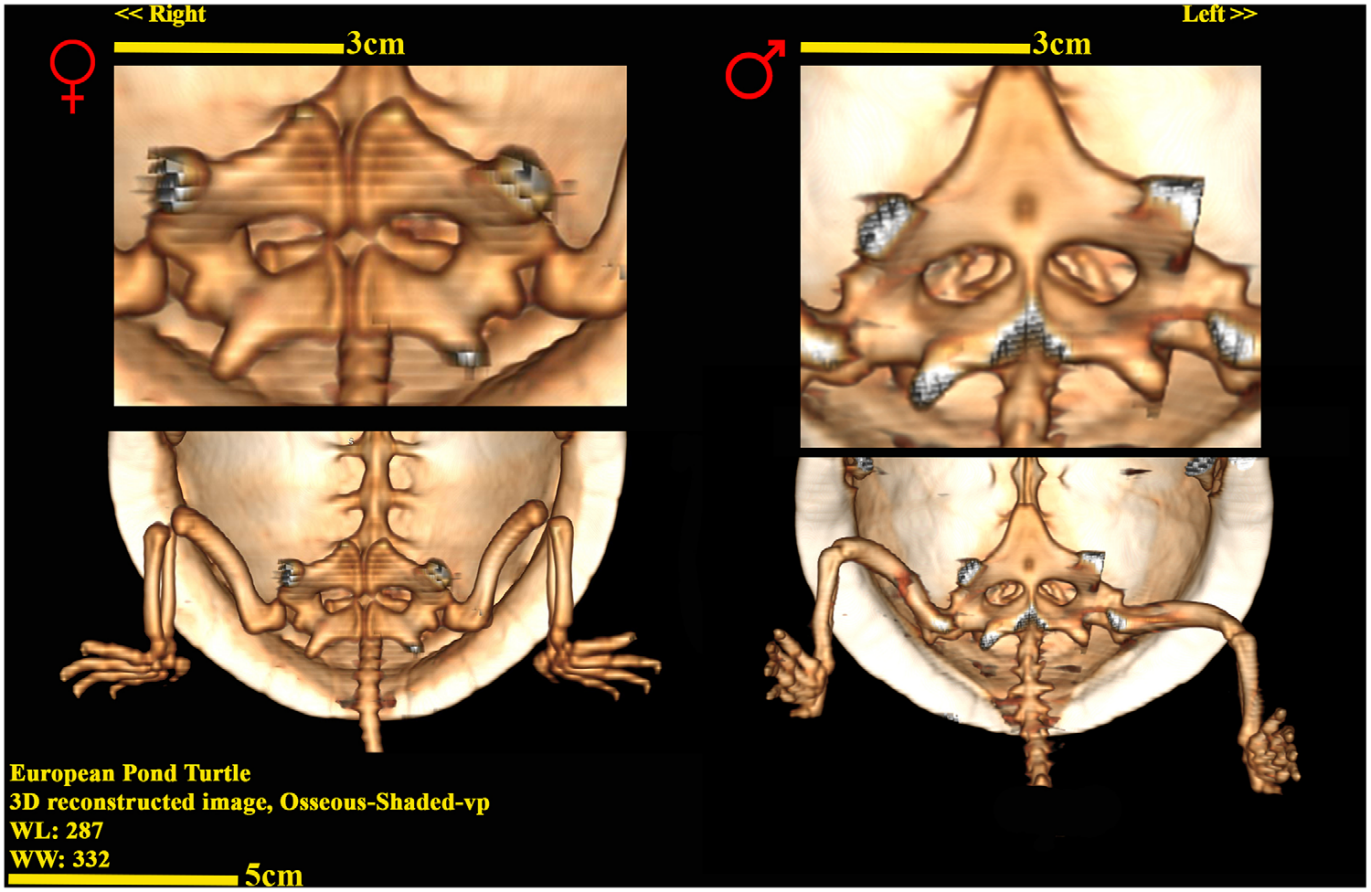
Ventral view of the skeleton of the male and female European pond turtle, 3D reconstructed CT scan image and osseous‐shaded‐vp pattern (The plastron has been removed).

Figure 16 provides a comprehensive view of the complete skeleton of the European pond turtle. During the examination of the specimens, several distinctions emerged between the hip bones of male and female turtles. In male turtles, a cranial ridge was noticeable along the medial aspect of the cranial edge of the pubic bone (Figure 16). It is noteworthy that in female turtles, this protrusion appeared considerably smaller. Furthermore, the pubic symphysis was predominantly ossified in male turtles, whereas it remained largely cartilaginous in female samples. It is important to note that these distinctions were evident in turtles that were all adults and within the same age range (Figure 17). Beyond the hip bone, no morphological variations were discerned between male and female turtles.

## 4 Discussion

Due to the relatively small dimensions of the manus and pes in this species, micro‐CT was employed to achieve a more precise morphological evaluation. This advanced imaging modality provided enhanced resolution, facilitating a detailed examination of the intricate bone structures (Zehtabvar, Masoudifard, et al. 2026).

Numerous studies have delved into the anatomy and radiological aspects of turtle bones, with notable works by Valente et al. in 2006 and 2007 (Valente et al. 2006, 2007). These studies have paid careful attention to device settings and the required kilovoltage for producing high‐quality radiographs in sea turtles. Valuable insights from these investigations suggest that adjusting kilovoltage is advisable, increasing it in the cranial third of the carapace length and decreasing it in the caudal third. Additionally, it has been proposed that the use of mammography films enhances the visibility of details in reptilian radiographs (Valente et al. 2006). In the present study, the utilization of CT scanning allowed for a more intricate examination of bones. Employing the 3D CT scanning technique mitigated issues associated with overlapping bones and other structures encountered in radiographic analysis, as previously noted in other studies (Asadi Ahranjani et al. 2016).

Valente et al., in their 2007 investigation of *C. caretta*, provided insights into the radiographic anatomy of the appendicular system, supplementing radiographs with 3D CT scan images (Valente et al. et al. 2007). Our study benefited significantly from 3D CT scan images, which facilitated the precise assessment of bone direction, angle and positioning. Notably, Asadi Ahranjani et al. (2016) highlighted challenges posed by the small size of certain bones and their loose interconnections, often leading to joint breakage during skeleton preparation. Consequently, the use of radiography and 3D CT scan images proved invaluable, particularly for reconstructing skeletons in exotic species with limited prior anatomical investigations (Asadi Ahranjani et al. 2016). In our examination of the European pond turtle, 3D CT scanning played a pivotal role in constructing a comprehensive skeleton.

The findings of Asadi Ahranjani et al. (2016) underscore distinctions in the appendicular limbs of the Euphrates softshell turtle (*R. euphraticus*) and sea turtles. These differences manifest in the general shape of bones, which markedly varies between the two species. These distinctions could be attributed to differences in movement behaviour and habitat, with sea turtles evolving into adept swimmers, leading to limb adaptations into fins. Conversely, the Euphrates softshell turtle exhibits unique characteristics, including protruding last phalanxes in the first three digits, accompanied by soft tissue and distinctive digital structures, reminiscent of fins. In contrast, the European pond turtle features distinct pes and manus digits with elongated and individualized structures. Remarkably, all the manus digits possess nails, and the digits exhibit elongation and distinct characteristics, void of fin‐like structures. This distinction may be attributed to the European pond turtle's dual habitat, as they inhabit both water and land. It is worth noting that the fifth pedal digit lacks a nail in this turtle species (Asadi Ahranjani et al. 2016).

The European pond turtle displays distinct anatomical characteristics compared to other turtle species, particularly aquatic ones. Notably, this turtle exhibits nails on all its manus digits, which are elongated and non‐fin‐like. This divergence in digit structure may be attributed to its dual habitat, as European pond turtles inhabit both aquatic and terrestrial environments. However, the fifth pedal digit lacks a nail in this species.

Contrasting with the Euphrates softshell turtle, where the pectoral girdle spans from the second to the sixth rib, the European pond turtle's pectoral girdle extends from the area just before the first rib to the third rib. Additionally, the orientation of the glenoid cavity differs: In the Euphrates softshell turtle, it faces cranially, whereas in the European pond turtle, it is located in a craniolateral position. In both species, the glenoid cavity takes on a bean‐shaped form. In the European pond turtle, the coracoid bone contributes to the ventral portion of the cavity, whereas in the Euphrates softshell turtle, part of the glenoid cavity created by the coracoid bone is positioned laterally.

Regarding the humerus, significant variations exist between species. In the Euphrates softshell turtle, it is elongated with distinct ends and a round body, whereas in sea turtles, it has a wide, flattened shape with a lateral projection (Wyneken 2001). The European pond turtle's humerus exhibits an S‐shaped curvature and body structure similar to the Euphrates softshell turtle, albeit lacking the groove observed in the latter. Additionally, the European pond turtle's humerus features lateral and medial borders, whereas the Euphrates softshell turtle's humerus has dorsal and ventral borders. The intertubercular fossa, observed in the proximal part of the humerus in the European pond turtle, also appears in the Euphrates softshell turtle but was not named in the study (Asadi Ahranjani et al. 2016).

In sea turtles, the radius bone is shorter than the ulna bone, whereas in the Euphrates softshell turtle and European pond turtle, the ulna bone is wider and shorter than the radius. Both the Euphrates softshell turtle and European pond turtle exhibit similarities in their forearm bone (radius and ulna) structures, including articulations with carpus bones. However, the European pond turtle's ulna also articulates with the pisiform bone, distinguishing it from the Euphrates softshell turtle. In terms of the wrist's proximal row, the Euphrates softshell turtle features the ulnar bone as the largest, whereas in the European pond turtle, it is the centrale bone. Both species possess five bones in their proximal carpus rows. Notably, the European pond turtle has a small sesamoid bone on the medial side of the centrale bone, absent in the Euphrates softshell turtle. Additionally, a 2019 study by Abdala et al. highlighted the presence of the pisiform and a small accessory element on the radial side of the carpal joint in matamata (*Chelus fimbriatus*), noting the absence of these structures in many other turtles (Abdala et al. 2019).

The differences between the European pond turtle (*E. orbicularis*) and other turtle species, such as the Euphrates softshell turtle and sea turtles, extend beyond limb anatomy and include pelvic girdle and hip bone characteristics.

In the European pond turtle, the centrale bone of the carpus is notably larger and occupies a substantial portion beneath both the radial and ulnar bones. Conversely, in the Euphrates softshell turtle, the centrale bone is relatively small and situated between the proximal and distal rows. Additionally, in the Euphrates softshell turtle, the first distal carpal bone articulates with the distal end of the radius, a feature not observed in the European pond turtle, where this bone articulates proximally with the centrale bone and the sesamoid bone adjacent to it. Metacarpal bone structures are generally similar in both species, with minor differences in the number of phalanges and the presence of unguals. Specifically, in the European pond turtle, all five digits have unguals, whereas the Euphrates softshell turtle exhibits varying numbers of phalanges and unguals across its digits. Additionally, the distinctive protrusion of the first phalanx on the proximal part of the palmar surface, seen in the European pond turtle, has not been reported in the Euphrates softshell turtle (Asadi Ahranjani et al. 2016).

Further differences are evident in the pelvic girdle and hip bones. In sea turtles, each hip bone features a hole, whereas in the Euphrates softshell turtle, only one hole is present in the entire pelvis. The pubic bone in sea turtles is relatively flat and uniform in width from cranial to caudal, whereas in the Euphrates softshell turtle, the pubic bone starts wide and gradually tapers into a shaft‐like structure. The ischium bones in sea turtles are rod‐shaped, with both bones connected to form a smooth caudal edge. In contrast, the Euphrates softshell turtle's ischium bone features a metaschial process and assumes a bow‐like shape.

The orientation of the acetabular cavity also varies between species. In sea turtles, the acetabular cavity is inclined laterally and caudally, whereas in the European pond turtle, it is inclined laterally and cranially. Additionally, although the European pond turtle exhibits large thyroid fenestrae on both the right and left sides of the pelvis, sea turtles typically have a single large thyroid fenestra.

The unique protrusion on the cranial edge of the pubic bone observed in the European pond turtle has not been reported in the Euphrates softshell turtle. However, both species share a common feature in the presence of a metischial process (Asadi Ahranjani et al. 2016).

The differences observed in the hip bones of male and female European pond turtles align with findings in Balkan terrapin (*Mauremys rivulata*) (Hussein Yousif 2022), indicating potential sexual dimorphism in these structures.

The comparison of femur bone characteristics between sea turtles, the Euphrates softshell turtle and the European pond turtle reveals notable distinctions among these species.

In sea turtles, the femur bone is relatively short and possesses an irregular geometric shape. Conversely, both the Euphrates softshell turtle and the European pond turtle feature elongated femurs with distinct extremities, similar to the humerus bone. The femur of the European pond turtle exhibits an elongated structure with a slight S‐shaped curve, mirroring the Euphrates softshell turtle. The femoral head in both species is oval. The overall structure of the femur is largely comparable, except for the groove observed in the distal part of the European pond turtle, which has not been reported in the Euphrates softshell turtle (Asadi Ahranjani et al. 2016).

In terms of trochanters, the European pond turtle features trochanters of nearly the same size and major and minor trochanters have not been named in this species. Conversely, in the Euphrates softshell turtle, specific trochanter names are used to describe the femoral structure (Asadi Ahranjani et al. 2016).

Another difference lies in the relative sizes of the articular surfaces on the distal end of the femur for articulation with the tibia and fibula. In the Euphrates softshell turtle, the articular surface of the tibia is larger than that of the fibula. In contrast, in the European pond turtle, these particular surfaces are almost equal in size (Asadi Ahranjani et al. 2016).

The comparison of the tibia bone between the Euphrates softshell turtle and the European pond turtle reveals distinct differences. In the Euphrates softshell turtle, the proximal end of the tibia bone is larger than the distal end. In contrast, the European pond turtle exhibits the opposite pattern, with the distal end of the tibia being larger than the proximal end (Asadi Ahranjani et al. 2016).

Overall, the limbs of turtles, including the arrangement and number of carpal and tarsal bones, have undergone significant evolutionary changes (Sánchez‐Villagra et al. 2007). These changes are so diverse that the anatomy of the autopodial region, particularly the digits and the tarsus and carpus, is utilized in the classification of early turtles. In the present study, the investigation and comparison of the European pond turtle's appendicular skeleton, particularly in the tarsus/carpus region, contribute to our understanding of the anatomical diversity among turtle species.

The number and shape of carpal and tarsal bones in the Spiny softshell turtle *(A. spinifera*) species are highly similar to each other. In *A. spinifera*, the formula for manual digits (digiti manus) is 2.3.3.4.3, and the formula for pedal digits (digiti pes) is 2.3.3.5.2. The primary difference between *A. spinifera* and the Euphrates softshell turtle lies in the number of phalanges in the fourth and fifth carpal and pedal digits (digiti pes).

In the Euphrates softshell turtle, these digits possess 5 and 4 phalanges in the manus and 4 and 3 phalanges in the pes, respectively. However, in *A. spinifera*, the number of phalanges in the manus is 3 and 4, and in the pes, it is 2 and 5. This discrepancy in the number of phalanges is a notable difference between these two species.

It is interesting to note that the fifth metatarsal in *A. spinifera* aligns with the distal tarsal bones and articulates with the fourth distal tarsal bone. This arrangement raises the question of whether the fifth distal tarsal bone was originally attached to the primary fifth metatarsal bone.

Sheil's findings in 2003, which investigated the development of bones during the embryonic period of *A. spinifera*, provide an answer to this question. According to Sheil's research, during the embryonic period, *A. spinifera* initially possesses four distal tarsal bones, and the fifth distal tarsal bone is absent. The fifth metatarsus, from the early stages of its formation, articulates with the fourth distal tarsal bone. This information sheds light on the developmental process of these bones in *A. spinifera* (Sheil 2003a).

In the European pond turtle, as mentioned above, the number of carpal bones in the proximal and distal rows is five each. In addition, there are five digits and five metacarpals in the manus, the way of their placement and the number of phalanxes is mentioned, which is somewhat different from the mentioned species.

In a study conducted on the Balkan pond turtle (*M. rivulata*), it is mentioned that this species has five digits in the manus region, and it is also mentioned that there are 10 carpal bones in total in this species (Hussein Yousif 2022). It should be noted that in the forelimb of the European pond turtle, digits number one and five have two phalanxes, and digits number two, three and four have three phalanxes, which of course, the same pattern is also observed in the hindlimb. In addition, in this species, both the forelimb and hindlimb have five digits. In the Balkan pond turtle, it is mentioned that the forelimb has five digits and the hindlimb has four digits (Hussein Yousif 2022).

The European pond turtle exhibits similarities in the overall structure of its tarsal and metatarsal bones when compared to the Euphrates softshell turtle. However, differences arise in the number of phalanges present in the digits of these two species.

In the hind limb of the Euphrates softshell turtle, the first, second, third, fourth and fifth digits have two, three, three, four and three phalanges, respectively. In contrast, the European pond turtle displays a distinct pattern, with the first and fifth digits possessing two phalanges each, whereas the second, third and fourth digits have three phalanges (Asadi Ahranjani et al. 2016).

One notable observation in the European pond turtle is the presence of sesamoid bones in its appendicular skeleton. This feature has not been reported in other species and might be attributed to the utilization of micro‐CT scan technology in this study, enabling a more detailed examination of the skeleton.

In the Euphrates softshell turtle, only the first, second and third pedal digits (digiti pes) have unguals, whereas in the European pond turtle, all the distal phalanges possess unguals, except for the fifth one (Asadi Ahranjani et al. 2016). There is no mention of the presence or absence of unguals in the context of the Balkan pond turtle (Hussein Yousif 2022).

When examining the number of tarsal bones in the *Chelydra serpentina* and Alligator snapping turtle (*Macrochelys temminckii*) species, it becomes evident that they possess six tarsal bones. These species also feature five distal tarsals, which is one more than observed in the Euphrates softshell turtle. Additionally, they have one bone in their proximal row, resulting from the fusion of the fibula, intermediate, proximal centrale and fourth centrale bones. Conversely, both the Euphrates softshell turtle and the European pond turtle possess only one bone in their proximal row, known as the *Astragalus* (talus) bone. The European pond turtle contains four independent bones in its distal row (Asadi Ahranjani et al. 2016).

It is worth noting that Arrau turtle (*Podocnemis expansa*), another species, has one more bone in each of its proximal and distal rows (Vieira et al. 2011).

The carpal digit formula for three species, *M. temminckii*, *C. serpentina* and *P. expansa*, is reported as 2.3.3.3.3 (from medial to lateral) (Vieira et al. 2011). These species differ from the Euphrates softshell turtle in their fourth and fifth digits, which have three phalanges each, whereas in the Euphrates softshell turtle, these digits possess five and four phalanges, respectively (Asadi Ahranjani et al. 2016).

In the European pond turtle, the digit formula for the Manual digits (digiti manus) is 2.3.3.3.2, with digits number one and five having two phalanges and digits number two, three and four having three phalanges. Notably, no sesamoid bones were observed in the vicinity of the phalanges. It is worth mentioning that this specific carpal digit formula has not been reported in other turtle species in various studies. However, the formula 2.3.3.3.3 has been reported in *Emydura subglobosa*, which is similar to this formula, with the only difference being that the fifth digit has three phalanges (Werneburg et al. 2009).

The tarsal digit formula in the two species, *M. temminckii* and *C. serpentine*, is reported as 2.3.3.3.2. The main difference between these species and the Euphrates softshell turtle is in the fourth and fifth digits, which have four and three phalanges, respectively, in the former two species, whereas in the Euphrates softshell turtle, these digits have three and two phalanges, respectively (Asadi Ahranjani et al. 2016).

In the European pond turtle, the digit formula for the pes (hindlimb) digits is 2.3.3.3.2, with digits number one and five having two phalanges, and digits number two, three and four having three phalanges each.

In another study by Sanchez‐Villagra et al. in 2007, they examined the autopodial skeleton in side‐necked turtles (*Pleurodira*) and found that there is more morphological variation in the carpal region compared to the tarsus. This variation includes differences in the connection of the distal carpals number 3, 4 and 5 or only 4 and 5, variations in the number of centrale bones, the presence or absence of the pisiform bone and differences in the connection or separation of the *Astragalus* (talus) and calcaneus bones in the tarsal area (Sánchez‐Villagra et al. 2007).

In the European pond turtle, the distal carpals are separate and articulate with other bones. This species also has a pisiform bone in the proximal carpal row and an *Astragalus* (talus) bone in the tarsus region, but it lacks a calcaneus bone in the proximal row.

In conclusion, the use of diagnostic imaging techniques, such as CT scans and micro‐CT scans, in the study of turtle skeletons has proven to be highly beneficial. These techniques enable researchers to accurately determine the direction and position of bones, even in species with complex anatomical features like turtles. Moreover, radiographic images and 3D CT scans are invaluable for reconstructing complete skeletons, particularly in the case of exotic species where detailed skeletal studies may be lacking.

Regarding why micro‐CT is used in this study, it should be noted that this technique is one of the new diagnostic imaging techniques that is rarely used in veterinary medicine, and due to its limitations (such as the size of the animal), its use in situations such as diagnosing diseases may be very limited and cannot be used in animals. However, due to its high accuracy and the fact that very small samples can be studied using this method, it is a very suitable technique for studying fine structures in gross anatomy, which is why this method was used in this study.

Regarding the limitations of this study, we should mention the following: limitation on the number of samples and low resolution in micro‐CT image reconstruction capabilities. This is clearly visible in the reconstructed 3D micro‐CT images of the manus and pes, and this problem could be due to errors in the imaging settings. Of course, it should be noted that in this area the structures are very delicate, and the skin, although thin, has structures that are scale‐like. The image of these structures also overlays the image of the phalanges and other bones.

Turtles present unique challenges due to their internal structures being enclosed within protective shells. Diagnostic imaging methods help overcome these limitations and facilitate the examination and understanding of turtle anatomy. These techniques are not only valuable for scientific research but also have important implications for conservation efforts. By using diagnostic imaging, anatomical studies can be conducted on living animals, reducing the need to sacrifice specimens, which is especially crucial for endangered species.

## Author Contributions

**Omid Zehtabvar**: conceptualization, investigation, funding acquisition, writing – original draft, methodology, validation, visualization, writing – review and editing, software, formal analysis, project administration, data curation, supervision, resources. **Ali Reza Vajhi**: conceptualization, investigation, methodology, software. **Somaye Davudypoor**: investigation. **Roshanak Mokhtari**: methodology, software. **Kiana Farahani**: methodology, formal analysis, data curation. **Seyyed Hossein Modarres Tonekabony**: investigation. All authors read and approved the final version of the manuscript.

## Funding

The authors have nothing to report.

## Ethics Statement

This study was continued of a doctor of veterinary medicine thesis and all experimental procedures were approved by the Faculty of Veterinary Medicine, University of Tehran Local Ethics Committee (ID: 601). It should be noted that no animal was euthanized for this study and all the samples recovered and were alive after CT scan. Turtle cadavers from Tehran veterinary clinics that died due to problems unrelated to the skeletal system were used for gross anatomy and micro‐CT scan studies.

## Conflicts of Interest

The authors declare no conflicts of interest.

## Supporting information




**Supporting File 1**: 3D Reconstructed clip of CT scan of complete skeleton, European Pond Turtle.

**Supporting File 2**: Reconstructed clip of Micro CT scan of fore limb, European Pond Turtle.

**Supporting File 3**: Reconstructed clip of Micro CT scan of hind limb, European Pond Turtle.

**Supporting File 4**: Movements in the European Pond Turtle (Emys orbicularis), The right manus is abnormal.

## Data Availability

The data that support the findings of this study are available from the corresponding author upon reasonable request.
